# Transport of intensity diffraction tomography with non-interferometric synthetic aperture for three-dimensional label-free microscopy

**DOI:** 10.1038/s41377-022-00815-7

**Published:** 2022-06-02

**Authors:** Jiaji Li, Ning Zhou, Jiasong Sun, Shun Zhou, Zhidong Bai, Linpeng Lu, Qian Chen, Chao Zuo

**Affiliations:** 1 School of Electronic and Optical Engineering, Nanjing University of Science and Technology, No. 200 Xiaolingwei Street, Nanjing, Jiangsu Province 210094 China; 2 Jiangsu Key Laboratory of Spectral Imaging & Intelligent Sense, Nanjing University of Science and Technology, Nanjing, Jiangsu Province 210094 China; 3 Smart Computational Imaging Laboratory (SCILab), Nanjing University of Science and Technology, Nanjing, Jiangsu Province 210094 China

**Keywords:** Transmission light microscopy, Imaging and sensing

## Abstract

We present a new label-free three-dimensional (3D) microscopy technique, termed transport of intensity diffraction tomography with non-interferometric synthetic aperture (TIDT-NSA). Without resorting to interferometric detection, TIDT-NSA retrieves the 3D refractive index (RI) distribution of biological specimens from 3D intensity-only measurements at various illumination angles, allowing incoherent-diffraction-limited quantitative 3D phase-contrast imaging. The unique combination of z-scanning the sample with illumination angle diversity in TIDT-NSA provides strong defocus phase contrast and better optical sectioning capabilities suitable for high-resolution tomography of thick biological samples. Based on an off-the-shelf bright-field microscope with a programmable light-emitting-diode (LED) illumination source, TIDT-NSA achieves an imaging resolution of 206 nm laterally and 520 nm axially with a high-NA oil immersion objective. We validate the 3D RI tomographic imaging performance on various unlabeled fixed and live samples, including human breast cancer cell lines MCF-7, human hepatocyte carcinoma cell lines HepG2, mouse macrophage cell lines RAW 264.7, *Caenorhabditis elegans* (*C. elegans*), and live Henrietta Lacks (HeLa) cells. These results establish TIDT-NSA as a new non-interferometric approach to optical diffraction tomography and 3D label-free microscopy, permitting quantitative characterization of cell morphology and time-dependent subcellular changes for widespread biological and medical applications.

## Introduction

Since its invention in the 1600s, the optical microscope has experienced continuous development and become an indispensable tool for the visualization of micro-scale objects with high resolution in biomedicine, chemistry, material science, electronics, and other various fields of forefront research and industrial applications^1^. Fluorescence microscopy, in which fluorescent molecules are used to light up target proteins in cells or cellular components, is possibly the most far-reaching development for the visualization of weakly absorptive biological samples. Over the past several decades, a variety of imaging modalities in fluorescence microscopy, e.g., wide-field^2,3^, confocal^4–6^, total internal reflection fluorescence^7,8^, two/multi-photon^9,10^, and light-sheet^11,12^ fluorescence microscopy techniques, were developed as powerful tools for probing very small signals and revealing three-dimensional (3D) structural and functional properties of fixed and living cells and tissues with high specificity. More recently, the development of new fluorescent molecular probes and single-molecule detection schemes opened up the possibility to observe the behavior of individual biomolecules sequentially instead of their ensemble averaging. Far-field super-resolution fluorescence techniques, including stimulated emission depletion microscopy (STED)^13,14^, (fluorescence) photo-activated localization microscopy (PALM)^15,16^, stochastic optical reconstruction microscopy (STORM)^17,18^, and structured illumination microscopy (SIM)^19,20^ have enabled the observation of nanoscale subcellular details well beyond the diffraction limit. However, these techniques are not appropriate for imaging non-fluorescent samples or visualizing cellular components that cannot be fluorescently targeted. In addition, the exogenous fluorescent agents could induce negative effects on cellular functions like cell viability, and the associated photobleaching prevents live-cell imaging over an extended period of time^21^.

On the other hand, phase-contrast microscopy exploits the refractive index (RI) as intrinsic optical imaging contrast for label-free imaging of biological samples without the use of exogenous labeling agents^22–24^. The introduction of optical interferometry and holography into microscopy made it possible to measure tiny phase differences induced by the specimens, facilitating the evolution of phase imaging techniques from qualitative observation to quantitative measurement^25–30^. By combining optical holography with computed tomography, through either object rotation or illumination scanning, various types of optical diffraction tomography (ODT)^31–37^ have been developed to infer the volumetric RI distribution of biological specimens, extending quantitative phase imaging (QPI) to three dimensions. In particular, ODT enables 3D label-free microscopy and has been successfully applied to investigate various types of biological specimens, including blood cells, neuron cells, cancer cells, and bacteria. Nevertheless, due to the temporally coherent illumination typically used, these coherent QPI and ODT methods suffer from speckle noise that prevents the formation of high-quality images. Moreover, most of them require a specialized interferometric setup with complicated beam scanning devices (e.g., scanning galvo-mirrors), hindering their widespread adoption in the biological and medical communities.

In this work, we present a new non-interferometric ODT approach, termed transport of intensity diffraction tomography with non-interferometric synthetic aperture (TIDT-NSA), which retrieves the 3D RI distribution of the sample from axial intensity stacks captured at different illumination angles. Our method can be viewed as an advanced extension of QPI based on the transport of intensity equation (TIE) to 3D RI diffraction tomography, with the intensity transport extended from “2D planar transport” to “3D volumetric transport”. Compared with computational optical sectioning (digital refocusing), the physical optical sectioning (through-focus scanning) in TIDT-NSA enhances the capability of scattering field collection at each different axial plane of thick samples, as a result of the combined effect of coherence and high numerical aperture (NA) gating. It has been found that the 3D intensity transport eliminates the need for the matched illumination condition (analyticity condition) as required in 2D Kramers-Kronig relations, and the resultant 3D Fourier spectrum of the intensity stack gives direct access to the object frequency content within the generalized aperture. A unified transfer function theory of 3D image formation is derived that relates the 3D object function (scattering potential) to the 3D intensity distribution under first-order Born/Rytov approximations, allowing for direct non-interferometric 3D synthetic aperture in the Fourier domain. Based on an off-the-shelf bright-field microscope equipped with a programmable light-emitting-diode (LED) array as the light source, we present high-resolution (330 nm/206 nm lateral resolution, 1.58 µm/0.52 µm axial resolution with dry/oil-immersion objectives, respectively) non-interferometric 3D RI reconstruction results of various samples, including human breast cancer cell line MCF-7, human hepatocyte carcinoma cell line HepG2, muscle cell culture murine skeletal myoblasts C2C12, mouse macrophage cell line RAW 264.7, and *Caenorhabditis elegans* (*C. elegans*). We further demonstrate long-term time-lapse 3D imaging of in vitro Henrietta Lacks (HeLa) cells, suggesting the developed TIDT-NSA is a promising, non-invasive tool for revealing morphology and dynamics of biological processes at cellular and subcellular levels.

## Results

### Unified theory for optical transfer functions and space-domain Kramers-Kronig relations

For QPI of 2D thin specimens, the object is described by a 2D complex amplitude function $O\left( {{{\mathbf{r}}}} \right) = A\left( {{{\mathbf{r}}}} \right)\exp \left[ {j\phi \left( {{{\mathbf{r}}}} \right)} \right]$, where *A*(**r**) and *ϕ*(**r**) represent the absorption and phase components of the object, respectively; **r** denotes the spatial coordinates. While for ODT of 3D thick specimens, the object is characterized by the complex scattering potential $O\left( {{{\mathbf{r}}}} \right) = k_0^2\left[ {n\left( {{{\mathbf{r}}}} \right)^2 - n_m^2} \right]$, where *n*(**r**) is the complex RI distribution of the object, *k*_0 _= 2*π*/*λ* is the wave vector in free space with *λ* being the illumination wavelength, and *n*_*m*_ is the RI of the surrounding medium. We use *a*(**r**) and *ϕ*(**r**) to represent the real and imaginary parts of the complex scattering potential, which correspond to the contributions of the absorption and phase components of the 3D object, respectively. It should be noted that we do not explicitly distinguish the space dimension within this paper, and the variable **r** represents 2D spatial coordinates (*x*,*y*) for the case of 2D QPI, and represents 3D spatial coordinates (*x*,*y*,*z*) = (**r**_*T*_,*z*) for the case of 3D ODT, with **r**_*T*_ representing the transverse spatial coordinates.

**Fig. 1:**
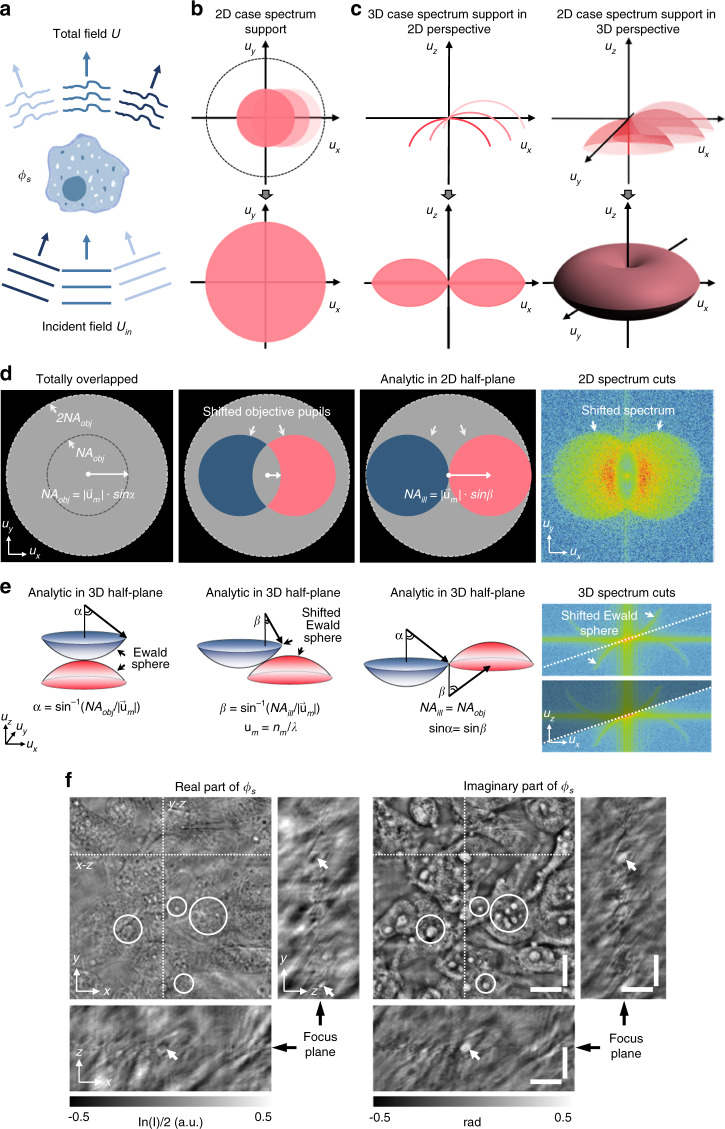
Illustration of the unified framework for 2D and 3D optical transfer functions and experimental demonstrations of 3D imaginary part recovery taking an example of unstained MCF-7 cells. **a** Object *ϕ*_*s*_ is illuminated by the plane waves from different directions, and the total field *U*(**r**) results from the interference between the scattered field *U*_*s*_(**r**) and the unperturbed fields *U*_*in*_(**r**). **b**, **c** Supports of frequency spectrum for the cases of 2D imaging and 3D imaging under angle-varied illuminations, and the full spectrum coverage for the complex amplitude of the 2D sample and the scattering potential of the 3D sample. **d**, **e** 2D and 3D phase transfer functions of system under different illumination conditions and experimental captured 2D and 3D frequency spectrum cuts. **f** Phase (imaginary) component of the 3D complex function is recovered from the 3D logarithm intensity stack (real part) via space-domain Kramers-Kronig relations. Scale bars: 10 µm.

Assuming that the sample is illuminated by a quasi-monochromatic plane wave with unit amplitude, the resultant total field *U*(**r**) can be regarded as the interferometric superposition of the incident field *U*_*in*_(**r**) and the scattered field *U*_*s*_(**r**) (Fig. 1a), i.e., $U\left( {{{\mathbf{r}}}} \right) = U_{in}\left( {{{\mathbf{r}}}} \right) + U_s\left( {{{\mathbf{r}}}} \right)$. Without loss of generality, we denote the contribution of the object as a complex phase function, which takes the form:

1 $$\begin{array}{l}\phi _s\left( {{{\mathbf{r}}}} \right) = \ln \left[ {U\left( {{{\mathbf{r}}}} \right)/U_{in}\left( {{{\mathbf{r}}}} \right)} \right] = \ln \left[ {1 + U_s\left( {{{\mathbf{r}}}} \right)/U_{in}\left( {{{\mathbf{r}}}} \right)} \right]\\\qquad\;\;\, \equiv a\left( {{{\mathbf{r}}}} \right) + j\phi \left( {{{\mathbf{r}}}} \right)\end{array}$$

For the case of 2D imaging, the significance of complex phase function is apparent: $\phi _s\left( {{{\mathbf{r}}}} \right) = a\left( {{{\mathbf{r}}}} \right) + j\phi \left( {{{\mathbf{r}}}} \right)$, where$a\left( {{{\mathbf{r}}}} \right) = \ln A\left( {{{\mathbf{r}}}} \right) = \ln I\left( {{{\mathbf{r}}}} \right)/2$ and *ϕ*(**r**) represent the real (absorption) and imaginary (phase) parts of the sample,$I\left( {{{\mathbf{r}}}} \right) = \left| {A\left( {{{\mathbf{r}}}} \right)} \right|^2{{{\mathrm{ = }}}}\exp \left[ {2{\mathrm{Re}} \left[ {\phi _s\left( {{{\mathbf{r}}}} \right)} \right]} \right]$ is the intensity that can be directly measured. As derived in Supplementary Information 1.1, under the first-order Born or Rytov approximation, a linearized relation between the first-order scattered field, $U_{s1}\left( {{{\mathbf{r}}}} \right) = U_{in}\left( {{{\mathbf{r}}}} \right)\phi _s\left( {{{\mathbf{r}}}} \right)$, and object function *O*(**r**) can be established for both 2D and 3D cases:

2 $${\left\{ {\begin{array}{*{20}{l}} {\hat O\left( {{{{\mathbf{u}}}} - {{{\mathbf{u}}}}_{in}} \right) = \hat U_{s1}\left( {{{\mathbf{u}}}} \right)P\left( {{{\mathbf{u}}}} \right)} & {{{{\mathrm{2D}}}}\,{{{\mathrm{sample}}}}} \\ {\hat O\left( {{{{\mathbf{u}}}} - {{{\mathbf{u}}}}_{in}} \right) = 4\pi ju_z\hat U_{s1}\left( {{{{\mathbf{u}}}}_T} \right)P\left( {{{{\mathbf{u}}}}_T} \right) \delta \left( {u_z - \sqrt {u_m^2 - \left| {{{{\mathbf{u}}}}_T} \right|^2} } \right)} & {{{{\mathrm{3D}}}}\,{{{\mathrm{sample}}}}} \end{array}} \right.}$$

where **u** is the spatial frequency coordinates corresponding to **r** [for 3D sample, **u** = (**u**_*T*_,*u*_*z*_) is the 3D spatial frequency coordinates], **u**_*in*_ is the incident frequency vector, $\hat O$ and $\hat U_{s1}$ represent the Fourier transforms of *O* and *U*_*s*1_, respectively (we use the “hat” to denote the signal spectrum in the 2D/3D Fourier domain). Note here *P* is the 2D complex pupil function of the imaging system [i.e., 2D coherent transfer function (CTF)], which ideally is a circ-function with a radius of *NA*_*obj*_/*λ*, determined by the objective NA. For the case of 2D samples, the first-order scattered field gives the information of the object spectrum within a shifted complex pupil function, as illustrated in Fig. 1b. For 3D samples, the 3D frequency vector **u** is located on the Ewald sphere with a radius of *u*_*m*_=*n*_*m*_/*λ* in 3D Fourier space under the constraint $u_z = \sqrt {u_m^2 - |{{{\mathbf{u}}}}_T|^2}$, and the 3D object frequency information accessible by the microscope is directly related to a subsection of the Ewald sphere (the projection of the 2D complex pupil function onto the Ewald sphere) so-called the generalized aperture $P({{{\mathbf{u}}}}) = P({{{\mathbf{u}}}}_T)\delta \left( {u_z - \sqrt {u_m^2 - |{{{\mathbf{u}}}}_T|^2} } \right)$ (i.e., 3D CTF), displaced by the incident frequency vector –**u**_*in*_ (see Fig. 1c).

The Fourier diffraction theorem (Eq. 2) suggests that each measurement of the first-order scattered field $\hat U_{s1}$ under a given illumination angle can only provide limited object frequency information determined by the illumination angle and the pupil function^38–40^. For the 2D case, single-angle measurement of $\hat U_{s1}$ can only deliver the object frequency content within the shifted pupil function. For the 3D case, a single-angle measurement of $\hat U_{s1}$ can only provide the object frequency content located on the shifted generalized aperture (a 2D partial spherical cap with no axial support). Consequently, one needs to illuminate the object from various directions, i.e., adjusting **u**_*in*_ to enlarge the accessible object spectrum, which allows the reconstruction of the complex amplitude of the 2D sample^41–44^ or the scattering potential of the 3D sample^45–51^ (Fig. 1b and c). For a typical microscopic system where the maximum illumination angle allowed is limited by *NA*_*obj*_, the maximum spectrum coverage is the same as that obtained in a conventional incoherent illuminated microscope, with a doubled lateral resolution (2*NA*_*obj*_/*λ*) compared to the coherent diffraction limit. For the 3D case, the maximum spectrum coverage forms a torus-shaped region of $\hat O\left( {{{\mathbf{u}}}} \right)$ with the half-side lateral and axial extensions of the frequency supports given by 2*NA*_*obj*_/*λ* and $\left( {n_m - \sqrt {n_m^2 - NA_{obj}^2} } \right)/\lambda$, as illustrated in Fig. 1c. In conventional QPI and ODT systems, the complex amplitude distribution (both amplitude and phase) of the total field $\hat U({{{\mathbf{u}}}}_T)$ is required to determine the first-order scattered field $\hat U_{s1}({{{\mathbf{u}}}}_T)$, which generally involves interferometric or holographic measurements. However, for non-interferometric QPI and ODT methods, one seeks to retrieve the phase or RI distribution of the sample from the intensity-only measurement of the total field

3 $$\begin{aligned} I\left( {{{\mathbf{r}}}} \right) =\, \left| {U\left( {{{\mathbf{r}}}} \right)} \right|^2 =& \left| {U_{in}\left( {{{\mathbf{r}}}} \right)} \right|^2\left| {\exp \phi _s\left( {{{\mathbf{r}}}} \right)} \right|^2 \\ = &\, \exp \, \left\{ {2{\mathrm{Re}} \left[ {\phi _s\left( {{{\mathbf{r}}}} \right)} \right]} \right\}\end{aligned}$$

Note that Eq. 3 is formally identical for both 2D and 3D cases. As derived in Supplementary Information 1.2, under the first-order Born or Rytov approximation, the logarithmic Fourier spectrum of the measured intensity imag e (or intensity stack for the 3D case) can be expressed as

4 $$\ln \hat I({{{\mathbf{u}}}}) = H_a({{{\mathbf{u}}}})\hat a({{{\mathbf{u}}}}) + H_\phi ({{{\mathbf{u}}}})\hat \phi ({{{\mathbf{u}}}})$$

where $\hat a({{{\mathbf{u}}}})$ and $\hat \phi ({{{\mathbf{u}}}})$ are the Fourier spectra of real (absorption) and imaginary (phase) parts of the object function *O*(**r**); *H*_*a*_(**u**) and *H*_*ϕ*_(**u**) are the corresponding amplitude transfer function (ATF) and phase transfer function (PTF), respectively:

5 $$\begin{array}{l}H_a\left( {{{\mathbf{u}}}} \right) = P({{{\mathbf{u}}}} + {{{\mathbf{u}}}}_{in}) + P^ \ast ({{{\mathbf{u}}}} - {{{\mathbf{u}}}}_{in})\\ H_\phi \left( {{{\mathbf{u}}}} \right) = P({{{\mathbf{u}}}} + {{{\mathbf{u}}}}_{in}) - P^ \ast ({{{\mathbf{u}}}} - {{{\mathbf{u}}}}_{in})\end{array}$$

where *P*(**u**) represents the 2D complex pupil or 3D generalized aperture function of the imaging system. Note that for 3D imaging, the factor 4*πju*_z_ in the second line of Eq. 2 should be incorporated into the the 3D generalized aperture. For 2D QPI and 3D ODT of unlabeled biological samples, the phase component $\hat \phi ({{{\mathbf{u}}}})$ dominates the intensity contrast and is also the quantity of interest. Figure 1d shows the 2D PTFs of an aberration-free imaging system under different illumination conditions. It can be found that for a perfectly in-focus imaging system, on-axis illumination (**u**_*in*_=0) produces no phase contrast because the two anti-symmetrical (positive and negative) pupils cancel each other out, suggesting that the phase structure cannot be observed in this case. Increasing the illumination angle ($0 < \left| {{{{\mathbf{u}}}}_{in}} \right| < NA_{obj}/\lambda$) makes the two pupils no longer completely overlap, thus rendering phase information visible. However, low-frequency phase components (near zero frequency) can be totally transferred if and only if the illumination angle matches the NA of the objective lens ($\left| {{{{\mathbf{u}}}}_{in}} \right| = NA_{obj}/\lambda$). Such a matched illumination condition is essential for accurate phase recovery based on asymmetric-illumination-based non-interferometric QPI methods, such as Fourier ptychographic microscopy (FPM)^52–56^ and differential phase contrast microscopy (DPC)^57,58^.

On a different note, the asymmetric-illumination-based non-interferometric QPI was recently interpreted from the perspective of space-domain Kramers-Kronig relations^59^. As discussed in Supplementary Information 2.1, the Kramers-Kronig relations describe the connection between the real and imaginary parts of a complex analytic function, which can be used to infer the phase component (imaginary) from the intensity (real) measurement The Titschmarch theorem states that a square-integrable function vanishing for a half-plane of the Fourier space results in its analyticity in the half-plane of the spatial domain^60^. For QPI of 2D thin specimens, it is easy to recognize that when the matched illumination condition is satisfied, the complex phase function *ϕ*_*s*_(**r**) is half-plane analytic, and its imaginary component *ϕ*(**r**) can be retrieved from its real component $a\left( {{{\mathbf{r}}}} \right) = \ln I\left( {{{\mathbf{r}}}} \right)/2$ via Hilbert transform from a single-shot intensity-only measurement (see Supplementary Information 2.2 for more details). Since the Hilbert transform can be concisely expressed as a half-plane ±*π*/2 phase filter in the Fourier domain^61^, the complex phase function *ϕ*_*s*_ can be alternatively retrieved via applying half-plane Fourier filtering on the logarithmic intensity spectrum equivalently. However, the matched illumination condition is difficult to strictly fulfill in practice, especially for high-NA microscopic systems (e.g., with an oil-immersion objective lens). The illumination generally cannot achieve the cutoff spatial frequency corresponding to *NA*_*obj*_, precluding intact recovery of phase component due to the low-frequency spectral overlapping in the captured intensity, or equivalently, the violation of analyticity in the corresponding complex phase function.

The situation will be completely different if we extend the intensity measurement into the 3D space. Similar to the 2D case, the 3D PTF also contains two anti-symmetrical generalized apertures shifted according to the incident illumination angle, as illustrated in Fig. 1e. However, because the two generalized apertures are defined and mirror-symmetrically shifted in 3D space, they never cancel each other out (except for the origin, which corresponds to the trivial constant phase), regardless of the illumination angle. The geometric structure of the 3D PTF inspires us to extend the Titschmarch theorem to 3D, exploiting 3D space-domain Kramers-Kronig relations to the 3D intensity (stack) instead of 2D intensity (image) to circumvent the restriction imposed by the matched illumination condition (Fig. 1e). As derived in Supplementary Information 2.3, the 3D complex phase function is always half-space analytic under arbitrary-angled illuminations, suggesting the imaginary component *ϕ*(**r**) can be retrieved from its real component $\ln I\left( {{{\mathbf{r}}}} \right)/2$ by simply taking 3D Hilbert transform (or 3D Fourier domain half-space filtering equivalently) on the measured 3D intensity stack. By further synthesizing the retrieved 3D complex phase functions at different illumination angles in the Fourier space, the object spectrum can be filled by the extracted generalized apertures, allowing for the reconstruction of the scattering potential of the 3D sample in a non-interferometric manner.

### Transport of intensity diffraction tomography with non-interferometric synthetic aperture

The unified theory for optical transfer functions and space-domain Kramers-Kronig relations reveals that the logarithmic intensity spectrum contains two conjugated terms sidebands akin to the “twin image” in the conventional holographic approach. In holography, the twin images can be separated for the off-axis geometry if the tilt angle between the object beam and the reference beam is large enough. While in the 2D case of non-interferometric measurement, the two conjugated terms can only be completely separated under the matched illumination condition. Otherwise, the low-frequency phase information in the spectrally overlapping region can never be recovered (Fig. 1d). Such a restriction does not exist for the 3D case, where the two conjugated Ewald spheres never intersect except for the origin (Fig. 1e). Figure 1f shows an example (MCF-7 cell) of the 3D complex phase distribution recovered from the 3D intensity stack by applying 3D half-space Fourier filtering on the logarithmic intensity spectrum to extract one Ewald sphere from the single-sideband spectrum when the matched illumination condition is not satisfied. It can be seen that the imaginary component of the 3D complex phase distribution is well recovered from the intensity stack, revealing important phase structures of the cell sample that are of low contrast and visibility in the real (absorption or amplitude) component. Physically speaking, the 3D complex phase function is a function of the 3D object rather than the 3D optical field, which only describes the overall contribution of the object to the incident illumination, i.e., the modulation effects on its amplitude and phase. It can be converted to the total field according to the relation $U\left( {{{\mathbf{r}}}} \right) = U_{in}\left( {{{\mathbf{r}}}} \right)\exp \left[ {\phi _s\left( {{{\mathbf{r}}}} \right)} \right]$, which propagates in 3D space with its intensity is the only measurable quantity for non-interferometric QPI and ODT methods (see Supplementary Information 5.1 for details).

**Fig. 2:**
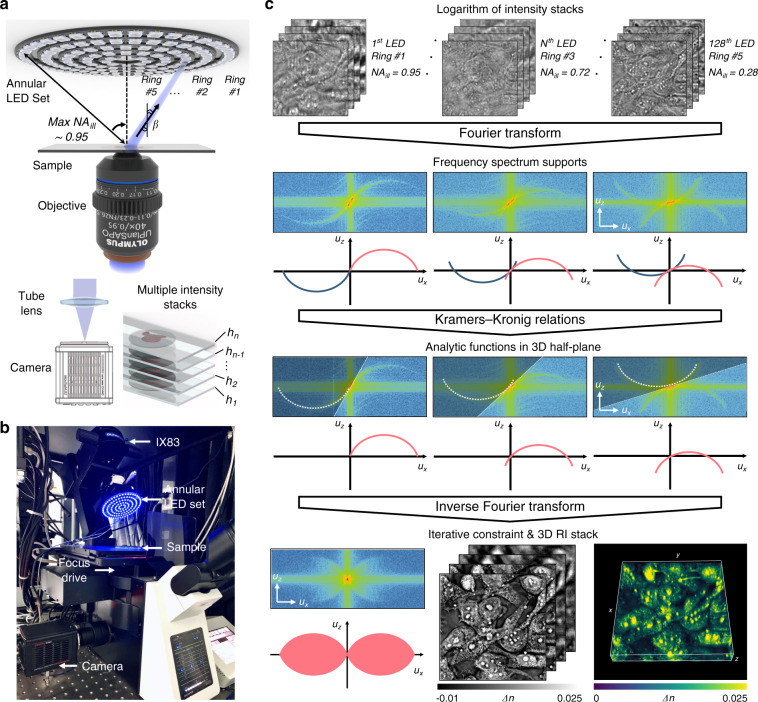
Hardware implementation and working flow of TIDT-NSA. **a** Setup of TIDT-NSA system based on a commercial microscope equipped with a multi-annular programmable LED source at the front-end of illumination and a scanning drive mechanism at the back-end of acquisition. **b** A photo of our TIDT-NSA hardware system. **c** Flowchart of data processing in TIDT-NSA for 3D RI reconstruction on the example of unstained MCF-7 cells.

Similar to conventional ODT methods, the 3D complex phase function recovered from a single illumination direction only occupies a very limited portion (located on a shifted Ewald sphere) of the 3D object spectrum; it has very poor depth discrimination or optical sectioning capability. To improve the resolution and reconstruct the scattering potential of the 3D sample, angle-scanning illuminations and synthetic aperture are further required to access an extended region of the 3D object spectrum. Thus, the proposed TIDT-NSA retrieves the 3D RI distribution of specimens through 3D synthetic aperture from 3D intensity measurements at different illumination angles. Due to the principle of non-interferometric measurement, TIDT-NSA can be easily implemented on an off-the-shelf bright-field microscope platform (IX83, Olympus, Japan) with the light source modified by a programmable multi-annular LED illumination, as shown in Fig. 2a. The programmable LED source is placed 25 mm above the sample, where 128 LED elements are used to produce narrow-band quasi-plane wave illuminations (central wavelength Red 629 nm and Blue 483 nm with 20 nm bandwidth) from different angles with a maximum illumination NA of 0.95. The 3D intensity stacks corresponding to different illumination angles are acquired by axial scanning the objective lens (UPLSAPO 40×, 0.95 NA, or UPLSAPO 100×, 1.4 NA, Olympus, Japan) utilizing a motorized focus drive with a minimum step size of 10 nm, and finally recorded by a scientific complementary metal-oxide-semiconductor (sCMOS) camera (ORCA Flash 4.0 V3 C13440, Hamamatsu, Japan, pixel resolution 2048 × 2048, pixel pitch 6.5 µm) under angle-varied LED illuminations. More details about the TIDT-NSA setup, imaging protocol, and system synchronization can be found in Supplementary Information 3.

Figure 2c illustrates the flowchart of data processing in TIDT-NSA for 3D RI reconstruction, taking unstained MCF-7 cells as an example. Following the idea of synthetic aperture, multiple through-focus intensity stacks are captured at different angled illuminations by turning on each LED element sequentially, and the corresponding 3D spectra can be obtained by taking 3D Fourier transform on the logarithmic intensity stack. As shown in the middle inset of Fig. 2c, two anti-symmetrical generalized apertures can be clearly observed in the *u*_*x*_–*u*_z_ cross-sections of the 3D spectra, showing good agreement with the theoretical distribution of 3D generalized aperture function corresponding to different illumination angles. After implementing the 3D half-space Fourier filtering on each dual sideband 3D spectrum (which is equivalent to applying the 3D space-domain Kramers-Kronig relations to the 3D intensity), the resultant spectra of 3D scattered field both containing real and imaginary parts of complex phase function are synthesized together in the Fourier space to get a preliminary estimate of the 3D object spectrum. Due to the discrete effect of the LED elements, the spectral sampling weights of different spatial frequencies of the synthesized 3D spectrum are not uniform. Moreover, the temporal and spatial coherence of the illumination (due to the finite wavelength bandwidth and size of the LED sources) results in spectrum spreading, making the detected Ewald sphere in each 3D logarithmic intensity spectrum “thicker” than that in the coherent case (see Supplementary Information 1.3 for detailed analysis). To compensate for these effects, 3D deconvolution is further performed on the preliminary synthesized spectrum based on a transfer function taking the LED discrete sampling, partial coherence of the illumination, and the correction factor (the 4*πju*_*z*_ factor in Eq. 2) into account. Moreover, a hybrid iterative constraint algorithm combining non-negative constraint and total variation regularization is used to computationally fill the missing cone information (the spectral region outside the frequency support). Finally, the scattering potential of the object is reconstructed after 3D inverse Fourier transform, and the corresponding 3D volumetric RI distribution can be used for label-free 3D imaging of biological samples, as shown in the bottom inset of Fig. 2c (see Video S1 and Video S2 for more experimental results of unstained MCF-7 cells). The detailed working flow of TIDT-NSA for 3D RI reconstruction can be found in Supplementary Information 4.

### Tomographic RI reconstruction of polystyrene beads

**Fig. 3:**
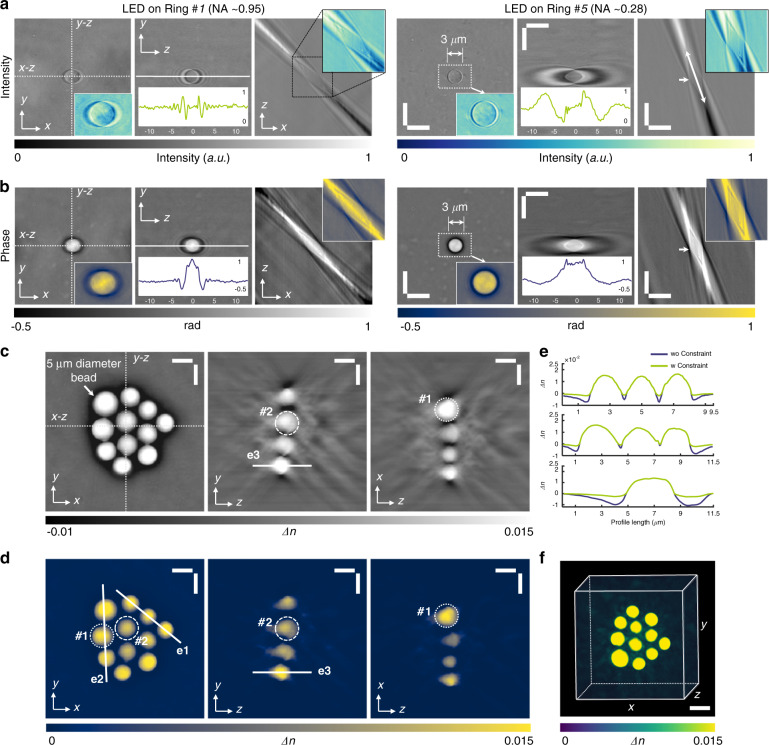
Experiments of 3D quantitative tomographic RI of polystyrene beads with different sizes. **a**, **b** Distributions of measured intensity stacks and retrieved phase stacks of micro polystyrene sphere with 3 µm diameter by implementing imaginary part recovery via intensity Kramers-Kronig relations under two different illumination angles. **c**–**e** RI tomograms of a cluster of polystyrene beads wo/w iterative constraint are reconstructed after the synthesis of the recovered 3D phase information under each illumination angle in the *x*−*y*, *x*−*z*, and *y*−*z* planes. **f** Rendered RI distribution as a 3D volume of bead cluster structure. Scale bars: 5 µm.

To validate the capability of TIDT-NSA for quantitative 3D RI reconstruction, a single polystyrene microsphere (Polysciences, calibrated *n* = 1.594 at *λ* = 483 nm) with 3 µm diameter and a mono-layer cluster of microspheres with different sizes immersed in RI matching oil (Cargille, *n*_*m*_ = 1.58) were imaged with a 100× oil-immersion objective lens. Figure 3a shows the *x–y*, *x–z*, and *y–z* slices of the 3D intensity distributions under two different illumination angles, and the double-headed arrow indicates the direction of incident illumination. It can be observed from the enlarged sub-regions that increasing the illumination angle slightly widens the lateral bead profile in the *x–y* plane but significantly shrinks the axial profile in the *y–z* plane, contributing to higher axial resolution and better depth sectioning. In contrast to conventional 2D bright-field imaging (focus plane indicated by the white arrows) where only high-frequency phase information can be revealed (since the illumination NA is insufficient to match the objective NA), as shown in *y–z* intensity slices of Fig. 3a, strong phase contrast can be observed in all 3D intensity distributions under arbitrary illumination angles, which demonstrates obvious axial anti-symmetry along the incident direction. By applying 3D half-space Fourier filtering (space-domain Kramers-Kronig relations equivalently) on each logarithmic 3D intensity spectrum, the corresponding 3D phase distributions (the imaginary part of the complex phase function) of the micro polystyrene sphere under different incident illuminations can be retrieved, as shown in Fig. 3b. The lateral spreading and axial elongation effects of the 3D phase distribution coincide well with those of the 3D intensity distribution. It should be noted that in conventional interferometric ODT methods, the 3D phase distributions can only be obtained through the numerical propagation of the total field $U\left( {{{\mathbf{r}}}} \right) = U_{in}\left( {{{\mathbf{r}}}} \right)\exp \left[ {\phi _s\left( {{{\mathbf{r}}}} \right)} \right]$ along the *z* direction once the phase of the total field is measured^32^, as discussed in Supplementary Information 2.2.

By synthesizing the recovered 3D phase distributions of each incident illumination, the RI tomograms of the bead cluster with different sizes were reconstructed (white arrow indicates 5 µm diameter bead), as shown in Fig. 3c. The RI slices are shown with a pixel spacing of 0.065 µm along all directions, and two beads in the cluster marked with dashed and dotted circles respectively are compared with their ideal circular shape. After applying the hybrid iterative constraint algorithm, the background artifacts due to incomplete angular coverage were eliminated, and the axial elongation of the reconstructed bead shape was mitigated, as shown in Fig. 3d. The reconstructed tomograms show a refractive index difference of Δ*n* = 0.0155 ± 0.0005 between the bead and its surroundings medium, in good agreement with the manufacturers’ specifications for beads and oil (Δ*n* = 0.0154). Finally, the rendered 3D volumetric RI distribution of the bead cluster was presented in Fig. 3f and animated in Video S3, demonstrating the feasibility and capability of the proposed TIDT-NSA for non-interferometric, accurate 3D RI reconstruction from intensity-only measurements.

It is worth noting that since the matched illumination condition cannot be satisfied in this oil-immersion imaging setting (illumination NA is much lower than objective NA), conventional asymmetric-illumination-based non-interferometric ODT methods, e.g., intensity diffraction tomography (IDT) and Fourier ptychographic diffraction tomography (FPDT) were unable to reconstruct the low-frequency RI components of the beads, as presented in Supplementary Information 5.2. These properties make TIDT-NSA more suitable than other non-interferometric ODT methods for imaging relatively thick samples such as the single-celled diatom microalgae presented in Supplementary Information 5.3. In Supplementary Information 6.1, we further tested the imaging resolution of the TIDT-NSA system by imaging both USAF and Siemens star targets, suggesting theoretical diffraction-limited 330 nm/206 nm lateral resolution and 1.58 µm/0.52 µm axial resolution with dry/oil-immersion objectives, respectively. Moreover, the sub-diffraction sized object (100 nm diameter nano-sphere) is utilized to demonstrate the ability of 3D RI tomography and the claim of achievable imaging resolution.

### Tomographic RI reconstruction of fixed biological samples

**Fig. 4:**
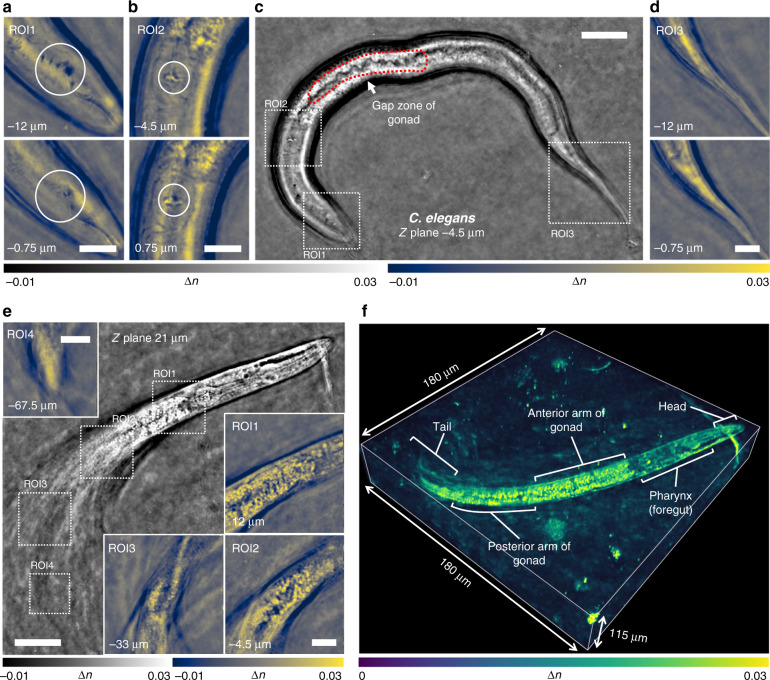
Tomographic RI reconstruction of fixed *C. elegans* at different stages of the life cycles. **a**–**e** Full FOV and different tomogram ROIs at different positions and axial planes to illustrate the recovered RI slice results of *C. elegans*. **f** 3D RI rendering of *C. elegans* worm over a volume of 180 µm × 180 µm × 115 µm. Scale bars: 25 µm (Full FOV) and 10 µm (ROIs).

Next, we applied TIDT-NSA to label-free 3D RI reconstruction of diverse fixed biological samples. To verify the capability of TIDT-NSA for imaging thick biological samples of strong scattering, the tomographic RI reconstructions of two fixed *C. elegans* worms at different stages of the life cycles are presented in Fig. 4. The sample was sandwiched between two coverslips, and the complete intensity stacks corresponding to all 128 illumination angles were acquired with a 40× objective for the tomographic RI reconstruction. The full lengths of the two juvenile *C. elegans* are ~250 µm, which were sufficient to be entirely imaged within a single field of view (FOV). As the upper worm was spread almost horizontally, the RI tomogram shown in Fig. 4c provides a morphological overview of the entire worm. Three different ROIs are further enlarged in Fig. 4a, b, and d to show the RI distribution of the *C. elegans* internal tissue structures at different depths from head to tail of the worm. Buccal cavity of *C. elegans* is clearly resolved in ROIs reconstruction as well as the corpus and grinder in terminal pharyngeal bulbs (Fig. 4a, large circles; Fig. 4b, small circles). The region enclosed by red dotted line indicates the gap zone of gonad, and this tomographic RI sections exhibits weaker RI contrast, as illustrated in Fig. 4c. The ROI slices in Fig. 4d show no reproductive system and a thin tail, suggesting this worm is more juvenile likely in the L3 or L4 development stage. In contrast, the other worm shown in Fig. 4e was more mature and distributed over a wider depth range (~100 µm). In addition, the posterior half of the worm body was highly twisted with the tail almost vertically arranged, which makes its tomographic reconstruction very challenging considering its large thickness, complex internal structures, and strong scattering properties. Figure 4f shows the full-volume RI reconstruction of the *C. elegans* worm over a volume of 180 µm × 180 µm × 115 µm (also see Video S8), in which the structures of the head, anterior arm of the gonad, posterior arm of the gonad, tail, and internal features are clearly resolved and comprehensively revealed. These results demonstrate the strong optical sectioning capabilities of TIDT-NSA for complex samples of strong scattering, benefited from the unique combination of through-focus scanning with illumination angle diversity. The high imaging accuracy and sensitivity of TIDT-NSA is evaluated in details in Supplementary Information 6.2. In Supplementary Information 7.1, we further demonstrate the label-free high-resolution 3D RI tomography capabilities of TIDT-NSA for imaging various fixed weakly scattering cell samples, including murine skeletal myoblasts cells C2C12, mouse macrophage cell lines RAW 264.7, and human hepatocyte carcinoma cell lines HepG2 (see also Video S4-Video S7).

### Dynamic 3D RI imaging of live cells

**Fig. 5:**
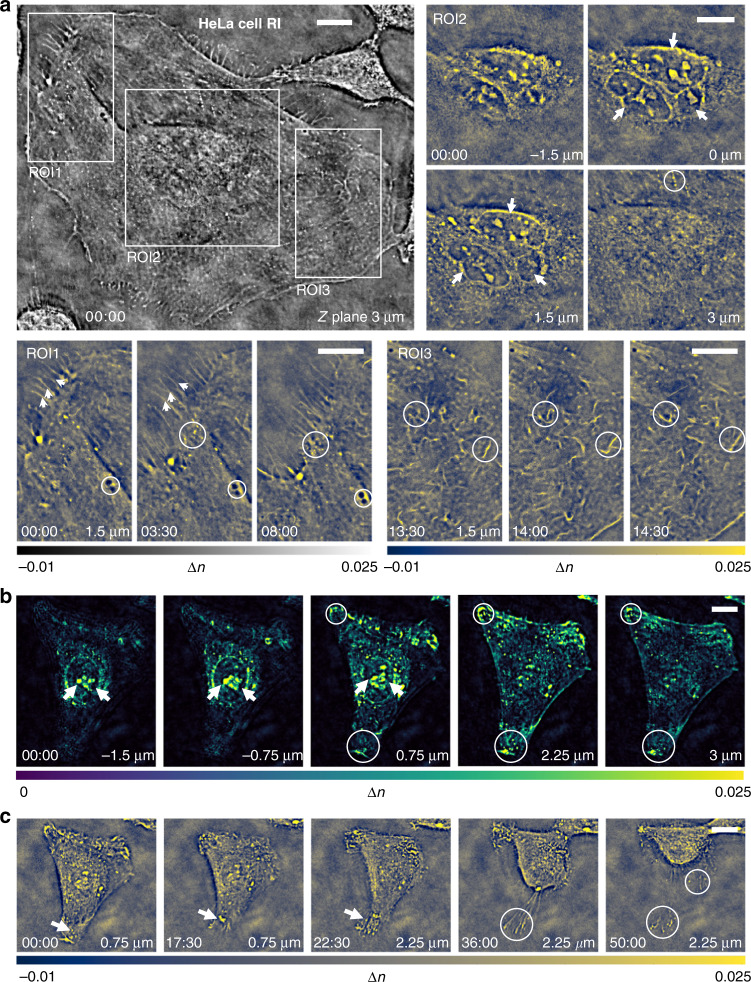
HeLa cells 3D RI imaging over hour-long time-lapse. **a** Recovered RI slice of trinucleated HeLa cell located at 3 µm Z plane at the start time point of 00:00:00, and enlarged time-lapse tomographic RI image of three different ROIs in the FOV. The whole process of HeLa cells visualization is given in Video S9. **b** Maximum intensity rendering of HeLa cell in another ROI located at different axial planes at the start time point of 00:00:00. **c** Cross-sectional view of the RI tomogram in the ROI at five different time points and axial planes. Scale bars: 15 µm.

The high-resolution 3D RI tomography capability of TIDT-NAS provides unique possibilities for the label-free time-lapse 3D imaging of live HeLa cells in culture. To accelerate the image acquisition process, 12 out of the total 128 LED elements were selected for sparser illumination sampling, and the number of axial layers of the intensity stack was also reduced to 17, resulting in a temporal imaging resolution of ~22 s per 3D frame (see subsection Imaging data processing and analysis and Supplementary Information 3.2 for more details). Figure 5a shows a cropped region containing a single widespread HeLa cell from the entire FOV of the reconstructed RI slice at *z* = 3 µm with a 40× objective, and three ROIs are zoomed to highlight subcellular details and dynamics at different depths. The multi-layer structure of three nucleoli in the trinucleate cell is revealed in *x–y* slices of the RI tomogram at different axial positions (arrows in ROI2). Two additional subregions highlight the dynamic processes of filopodia with high resolution and high RI contrast (arrows in ROI1), the rapid transport of tiny dark vesicle structures in the cytoplasm (circles in ROI2), and the fast motion of mitochondrial structures (circles in ROI3) with high resolution and high RI contrast. The corresponding time-evolution video of the RI tomograms of the live HeLa cell is provided in Video S9.

Figure 5b and c further provide the dynamic 3D RI imaging results of another HeLa cell, including the rendered RI slices at different axial planes and the RI slices at different time points, respectively. At the beginning of the observation, the nucleoli inside the cellular nucleus have higher RI values compared to the average index of the cytoplasm (Fig. 5b, arrows), and many small cytoplasmic particles with high RI, which may be lipid droplets, lysosomes, vesicles, or other organelles, were also clearly observed inside the cell. Also, at the 22-min time point, the cell began to contract, and the filopodia moved upwards with an obvious increase in its RI contrast, but the filopodia tips still adhered to the substrate of glass slip (arrows, Fig. 5c). After 30 min, the cell rounded up and organized its chromatin, leaving some filopodia tips still attached to the original position (Fig. 5c, circles) (see Video S10 for the corresponding time-evolution video of the RI tomograms). In Supplementary Information 7.2 and Video S11, we further provide the dynamic imaging results of an apoptotic HeLa cell. Major hallmarks of apoptosis, such as nucleus condensation, cytoplasm granulation, and blebbing, are clearly visualized in the time-lapse RI slices in both high resolution and negligible motion blur. Moreover, the related discussions about the possible photodamage of the illumination and quantitative analysis on the imaging resolution of living biological samples are detailed in Supplementary Information 7.2. These results demonstrate that TIDT-NSA is capable of observing unlabeled live cells in the traditional environment of an inverted microscope, allowing for high-resolution high-contrast 3D microscopy over an extended period of time.

## Discussion

In summary, we have developed TIDT-NSA, a new 3D microscopy technique for quantitative RI tomography of unlabeled specimens. By incorporating a programmable LED illumination unit with a standard bright-field microscope, we capture a series of 3D intensity stacks at various illumination angles and realize non-interferometric optical diffraction tomography utilizing 3D space-domain Kramers-Kronig relations. A unified transfer function theory of 3D image formation is derived that relates the 3D scattering potential to the 3D intensity distribution under first-order Born/Rytov approximations, revealing that the conjugated terms in the logarithmic intensity spectrum are always separable in the 3D Fourier space. TIDT-NSA eliminates the need for the matched illumination condition (analyticity condition) as required in 2D Kramers-Kronig relations, allowing for direct non-interferometric 3D synthetic aperture in the Fourier domain. Experiential results on various types of samples, including polystyrene beads, *C. elegans*, fixed and live cells, verified the high-resolution 3D imaging and optical sectioning capabilities of TIDT-NSA. In light of the distinguishing features presented herein, it is illustrative to consider a brief comparison of TIDT-NSA and other state-of-the-arts to elucidate their advantages and limitations, as summarized in Supplementary Information 2.4 and Table S1. It is clear from this table that the TIDT-NSA method addresses nearly all of the major limitations of the other methods.

However, some important issues still need to be clarified or require further investigation. First, despite its strong optical sectioning capabilities offered by the combination of z-scanning the sample with illumination angle diversity, TIDT-NSA still relies on the first-order Born or Rytov approximations and, thus, may not be applied well to multi-layer or multiple-scattering samples^62–64^. Second, due to the relatively large data amount requirement for both axial and angular scanning (at least 204 images in per 4D intensity stacks dataset), TIDT-NSA has not yet been fully optimized in acquisition speed, which is mainly limited by the z-scanning mechanics. There is still plenty of room for speed improvement, if a spatial light modulator^65,66^ or an electrically tunable lens^67^ is used to replace the scanning mechanics. Finally, due to the lacking of specificity, the fluorescence-assisted TIDT-NSA will be envisioned collaborating the advantages of specificity of fluorescence techniques and non-invasiveness of diffraction tomography, providing wider window and more insights to investigate biological processes^68,69^.

## Materials and methods

### Bead mounting and cell fixation

To prepare polystyrene bead samples, the mixture of micro polystyrene beads (Polysciences Inc., *n* = 1.594 at *λ* = 483 nm) with different diameters (mainly 3 and 5 µm) were diluted 1:100 in Millipore water and immersed in RI matching oil (E High Dispersion Series, *n*_*m*_ = 1.58, Cargille) followed by 1:100 dilution after drying. 100–200 µL of this mixture were dispersed and mounted on a coverslip, and the edge of coverslip (22 × 22 mm^2^, thickness 0.13 mm, catalog No. 12–545 F, Fisherbrand) was glued with cyanoacrylate (PT09, Pacer Technology, California) onto a 2.5 × 7.5 cm^2^ microscope glass slide (catalog No. 12-553-5B, Fisherbrand). The bead sample was pre-placed for two hours to avoid the effect of Brownian motion.

For unstained cell fixation (including HepG2 and RAW 264.7), cells were seeded in Lab-Tek II chambered cover slides (Nunc, Thermo Fisher Scientific) with a solution composed of 15% (v/v) fetal bovine serum (FBS), 100 U mL^−1^ penicillin, 100 µg mL^−1^ treptomycin, 20 mg L^−1^ gentamicin, 1 ng L^−1^ fibroblast growth factor, and 3 g L^−1^ sodium bicarbonate in Alpha-MEM (Minimum Essential Medium Eagle-Alpha Modification) media (all GIBCO, Thermo Fisher Scientific). At approximate 50% confluence, cells were washed twice in pre-warmed stabilizing buffer (MTSB, 0.1 M PIPES [pH 6.8], 4 M glycerol, 1 mM MgCl_2_, 2 mM EGTA, 0.1 mM EDTA), followed by application of pre-warmed fixation buffer (MTSB with 3.7% paraformaldehyde) for 10 min at room temperature. Cells were then washed three times for 5 min each with 1× PBS and DI-water, and the fixed cells were sealed between the coverslip and one microscope slide for phase imaging. The coverslip is pre-cleaned for cell imaging: we immersed the coverslips in 10% powdered precision ultrasonic cleaner (Branson, B200) and sonicated the coverslips for 10 min. After rinsing in deionized water, the coverslips were sonicated in acetone for 15 min, and then sonicated again in 1 M NaOH or KOH for 10 min. Finally, we rinsed the coverslips with deionized water, followed by sonication three times for at least 5 min each time. The washed coverslips were stored in an alcohol solution with a concentration >95% at 4 °C.

### *C. elegans* and HeLa cell culture preparation

The *C. elegans* were cultured on *E. coli* OP50-seeded Petri dishes (catalog No. AS4052, Fisher-brand) and maintained in an incubator at 20 °C. To immobilize worms, we transfer molten agarose (A6013 Type I, low EEO, Sigma-Aldrich) to slide containing plastic spacers followed by another glass slide to form a pad. After the preparation of the agarose pad has cooled, we transfer one or more NGM-washed *C. elegans* to a prepared pad containing 5–10% agarose in NGM buffer. A washed coverslip (thickness ~0.13 mm, catalog No. 12–545F, Fisherbrand) is placed on a glass slide, and the *C. elegans* worms are ready for imaging with a high numerical aperture objective.

HeLa cells were seeded (at an initial density of 300 cells/cm^2^) in a 35 mm glass-bottom Petri dish (No.0 Uncoated Coverslip, 10 mm Glass Diameter, MatTek Corporation, P35G010C) using DMEM high glucose with pyruvate (4.5 g 1:1 glucose, with GlutaMAX supplement, Gibco, Thermo Fisher Scientific or Roti-CELL DMEM, Roth) supplemented with 10% fetal bovine serum and 1× penicillin-streptomycin (both Gibco, Thermo Fisher Scientific). Cells were incubated at 37 °C in humidified atmosphere of 5% carbon dioxide for 8 h to allow attachment. After that, cells were washed twice with PBS, and pre-warmed fresh medium was added. Then, the cells were placed on the stage-mounted climate chamber (Tokai Hit INUF-IX3W, Japan) of the microscope for hour-long time-lapse dynamic-cell imaging.

### Imaging data processing and analysis

The data processing comprised several iterative steps, as summarized in Supplementary Information 4. First, individual 4D intensity stacks were correctly reordered under precise synchronization between camera, focus drive, and illumination sets. Second, implementing Kramers-Kronig relations on the logarithm of each intensity stack under each LED illumination for phase recovery and angle-varied 3D complex phase functions was obtained. Third, for the synthesis of the object’s scattering potential, individual aperture spectra were stitched; that is, caps of the Ewald sphere were used to sample a volume in the 3D spatial frequency domain. Fourth, the realistically inverse filtered spectra were realized by complex regularized 3D incoherent deconvolution, and the scattering potential of a 3D object is reconstructed.

For experiments of fixed samples (C2C12, MCF-7, RAW 264.7, and *C. elegans*), in total, 25728 intensity frames are acquired within a 4D intensity set (2048 × 2048 × 201 × 128) for 201 axial *z* slices, and 128 LED illuminations under dry objective over 1.5-h acquisition time (200 ms exposure time for each frame). The control bead sample and HepG2 cells are measured under an oil-immersion objective with the same illumination geometry and acquisition protocol. While for dynamic HeLa time-lapse imaging, the number of LED illuminations is down-sampled and the slices of axial step are also reduced to achieve the limit of system, and the 4D intensity set containing 12 stacks each with 17 axial frames (2048 × 2048 × 17 × 12) are captured and transferred within 22 s (50 ms exposure time for each frame under 40× objective).

Image analysis and processing were performed with Fiji and MATLAB (Mathworks). All other images were rendered using ImageJ, MATLAB (Mathworks), ICY., and Illustrator (Adobe) were used to analyze the data and to prepare the final images. Image processing was performed on a Windows 10-based workstation equipped with Intel Core i7-7820X processor operating at 3.6 GHz with eight cores and 16 threads, 128 GB of 2133 MHz DDR4 RAM, and NVIDIA GeForce GTX 2080Ti 11 GB graphics card, and the time required for a complete 3D reconstruction computation processes (including all 3D Fourier transform operations and once incoherent deconvolution operations) of intensity stacks full FOV (2048 × 2048 × 17 × 12) is about 9.6 s, which could be further reduced by using graphics processing unit parallel acceleration.

## Supplementary information

Supp

Video S1

Video S2

Video S3

Video S4

Video S5

Video S6

Video S7

Video S8

Video S9

Video S10

Video S11

## Data Availability

All data are available in the main text or the supplementary materials from the corresponding author upon reasonable request.
