# Supplementary material for: Transport of intensity diffraction tomography with non-interferometric synthetic aperture for three-dimensional label-free microscopy
Source: Light Sci Appl. 2022 Jun 2;11:154. doi: 10.1038/s41377-022-00815-7 (PMC9160286; doi:10.1038/s41377-022-00815-7)
Supplement: Supplementary file 1 — Supp [file 41377_2022_815_MOESM1_ESM.pdf]

# Supplementary Information for
# Transport of intensity diffraction tomography with non-interferometric synthetic aperture for three-dimensional label-free microscopy

**Jiaji Li[1,2,3], Ning Zhou[1,2,3], Jiasong Sun[1,2,3], Shun Zhou[1,2,3], Zhidong Bai[1,2,3], Linpeng Lu[1,2,3], Qian Chen[1,2,*], and Chao Zuo[1,2,3,**]**

[1]School of Electronic and Optical Engineering, Nanjing University of Science and Technology, No. 200 Xiaolingwei Street, Nanjing, Jiangsu Province 210094, China
[2]Jiangsu Key Laboratory of Spectral Imaging & Intelligent Sense, Nanjing University of Science and Technology, Nanjing, Jiangsu Province 210094, China
[3]Smart Computational Imaging Laboratory (SCILab), Nanjing University of Science and Technology, Nanjing, Jiangsu Province 210094, China
[*]chenqian@njust.edu.cn
[**]zuochao@njust.edu.cn

## ABSTRACT

This document provides supplementary information to “Transport of intensity diffraction tomography with non-interferometric synthetic aperture for three-dimensional label-free microscopy”.

**Correspondence to:** Chao Zuo, zuochao@njust.edu.cn; Qian Chen, chenqian@njust.edu.cn
**Website:** http://www.scilaboratory.com/

## Contents

**1. Transfer function theory for diffraction tomography**
**2. Kramers-Kronig relations in diffraction tomography**
**3. Hardware implementation for TIDT-NSA**
**4. TIDT-NSA reconstruction algorithm**
**5. Validation of TIDT-NSA on both simulations and experiments**
**6. Quantification and analysis of TIDT-NSA**
**7. Supplementary experimental results of biological cells**
**8. References**
**9. Supplementary Table**
**10. Supplementary Figures**
**11. Supplementary Videos**

# 1 Transfer function theory for diffraction tomography

## 1.1 Transfer function theory for holographic diffraction tomography

In optical diffraction tomography (ODT), the physical quantity of interest to be retrieved is the scattering potential of a thick three-dimensional (3D) sample, which is defined as

$$O(\mathbf{r}) = k_0^2 \left[ n(\mathbf{r})^2 - n_m^2 \right] \tag{S1}$$

where $k_0 = 2\pi/\lambda$ is the wave-number in free space, $\lambda$ is the illumination wavelength, $n_m$ is the complex refractive index (RI) of the surrounding medium, $n(\mathbf{r})$ is the spatial RI distribution of the sample, and $\mathbf{r} \equiv (x, y, z) \equiv (\mathbf{r}_T, z)$ is a short-hand notation for the 3D spatial coordinate. When a 3D sample is illuminated by a plane wave $U_{in}(\mathbf{r})$, the scattered complex field $U_s(\mathbf{r})$ obeys the following inhomogeneous wave equation:

$$\left(\nabla^2 + k_m^2\right) U_s(\mathbf{r}) = -O(\mathbf{r}) U(\mathbf{r}) \tag{S2}$$

where $k_m = k_0 n_m$ is the wave-number in the surrounding medium, $\nabla^2 = \frac{\partial^2}{\partial x^2} + \frac{\partial^2}{\partial y^2} + \frac{\partial^2}{\partial z^2}$ is the 3D Laplacian operator, and $U(\mathbf{r})$ is the total field that is written as the superposition of the incident (un-scattered) field, $U_{in}(\mathbf{r})$, and the scattered field, $U_s(\mathbf{r})$ :

$$U(\mathbf{r}) = U_{in}(\mathbf{r}) + U_s(\mathbf{r}) \tag{S3}$$

Using the Green's function method, we obtain the following relation, known as the Fourier diffraction theorem[1], which relates the first-order scattered field at the detection plane $(z = z_D)$ to the scattering potential in the Fourier space

$$\hat{O}(\mathbf{u} - \mathbf{u}_{in}) = 4\pi j u_z e^{-i2\pi u_z z_D} \hat{U}_{s1}(\mathbf{u}_T; z = z_D) \delta\left(u_z - \sqrt{u_m^2 - |\mathbf{u}_T|^2}\right) \tag{S4}$$

where $j$ is the imaginary unit, $\mathbf{u}_{in}$ is the 3D frequency vector of the incident plane wave, $\hat{O}(\mathbf{u})$ and $\hat{U}_{s1}(\mathbf{u}_T; z = z_D)$ are the 3D and 2D Fourier transforms of $\hat{O}(\mathbf{r})$ and $\hat{U}_{s1}(\mathbf{r}_T; z = z_D)$, respectively (we use the "hat" to denote the signal spectrum in the 2D/3D Fourier domain). $\mathbf{u} - \mathbf{u}_{in}$ denotes the shifted spatial frequency vector governed by the Lauer equation[2]. The exponential term in Eq. (S4), $e^{-i2\pi u_z z_D}$, can be interpreted as the angular spectrum propagation kernel for a coherent complex field, which accounts for the coordinate shift in the $z$ direction[3]. Note that this exponential term will automatically vanish if the measurement is performed at the nominal 'in-focus' plane ($z = 0$), and we neglect this term for simplicity:

$$\hat{O}(\mathbf{u} - \mathbf{u}_{in}) = 4\pi j u_z \hat{U}_{s1}(\mathbf{u}_T) \delta\left(u_z - \sqrt{u_m^2 - |\mathbf{u}_T|^2}\right) \tag{S5}$$

Because the 3D spatial-frequency vector, $\mathbf{u} = (\mathbf{u}_T, u_z)$, lies on the Ewald sphere under the constraint $u_z = \sqrt{u_m^2 - |\mathbf{u}_T|^2}$, the information defined by $\hat{U}_{s1}(\mathbf{u}_T)$, is directly related to a particular semi-spherical surface with a radius of the Ewald sphere $u_m = n_m/\lambda$ in the 3D Fourier space that is displaced by $-\mathbf{u}_{in}$.

However, for a practical microscopic system, only forward propagating waves falling within the system aperture can contribute to the image formation. For 2D imaging of a weakly scattering thin sample, the effect of the lens aperture is usually described by the 2D complex pupil function [i.e., coherent transfer function (CTF)] $P(\mathbf{u}_T)$, which ideally is a circ-function with a radius of $NA_{obj}/\lambda$, determined by the NA of the objective. Under oblique illuminations, the object spectrum is shifted and limited by the complex pupil function, $\hat{O}(\mathbf{u}-\mathbf{u}_{in}) = \hat{U}_{s1}(\mathbf{u})P(\mathbf{u})$[4,5]. For the case of 3D imaging of a thick sample, the complex pupil function should be projected onto the spherical surface, i.e., $P(\mathbf{u}) = P(\mathbf{u}_T)\,\delta\left(u_z - \sqrt{u_m^2 - |\mathbf{u}_T|^2}\right)$, resulting a subsection of the Ewald sphere called the generalized aperture (i.e., 3D CTF)[5]. Limited by the generalized aperture, Eq. (S5) should be modified as

$$\hat{O}(\mathbf{u}-\mathbf{u}_{in}) = 4\pi j u_z \hat{U}_{s1}(\mathbf{u}_T)P(\mathbf{u}_T)\,\delta\left(u_z - \sqrt{u_m^2 - |\mathbf{u}_T|^2}\right) \tag{S6}$$

Considering a practical imaging system with aberrations, the 3D CTF can be experimentally calibrated by fabricating a nanoscale object to simulate a complex infinitesimal point source in the 3D space[6]. As is clear here, the solution to the inverse scattering problem requires the first-order scattered field $U_{s1}(\mathbf{r}_T)$ either to be a measurable quantity or can be obtained by other means. Two approximations are often used to determine $U_{s1}(\mathbf{r}_T)$: The first-order Born approximation assumes that the objects are weak scattering $[U(\mathbf{r}_T) \approx U_{in}(\mathbf{r}_T) \gg U_s(\mathbf{r}_T)]$ so that $U_s(\mathbf{r}_T) \approx U_{s1}(\mathbf{r}_T)$. It is valid when the absorption and total phase delay introduced by the object is small. The first-order Rytov approximation assumes that the total field has a complex phase function related to the scattered field, i.e., $U(\mathbf{r}_T) = U_{in}(\mathbf{r}_T)\exp[\phi_s(\mathbf{r}_T)]$. When the phase gradient introduced by the object is small, $U_{s1}(\mathbf{r}_T) = U_{in}(\mathbf{r}_T)\phi_s(\mathbf{r}_T)$. The two approximations for $U_{s1}(\mathbf{r}_T)$ are summarized in Eq. (S7)[3].

$$U_{s1}(\mathbf{r}_T) \approx \begin{cases} U_s(\mathbf{r}_T) = U(\mathbf{r}_T) - U_{in}(\mathbf{r}_T) & \textit{Born approximation} \\ U_{in}(\mathbf{r}_T)\ln\left[\frac{U(\mathbf{r}_T)}{U_{in}(\mathbf{r}_T)}\right] = U_{in}(\mathbf{r}_T)\ln\left[\frac{U_s(\mathbf{r}_T)+U_{in}(\mathbf{r}_T)}{U_{in}(\mathbf{r}_T)}\right] & \textit{Rytov approximation} \end{cases} \tag{S7}$$

It should be noted that the Rytov approximation can be reduced to Born approximation when the scattered field is weak or the total phase delay induced by the object is small[5]. Therefore, for weakly scattering samples satisfying the Born approximation, $U_s \approx U_{s1} \ll U_{in}$, the complex phase representation is also valid, resulting in the following unified relation to the first-order scattered field:

$$U_{s1}(\mathbf{r}) = U_{in}(\mathbf{r})\,\phi_s(\mathbf{r}) \tag{S8}$$

The Fourier diffraction theorem (Fig. S6) suggests that for each illumination angle, only partial spherical cap bounded by the generalized aperture can be probed. Illuminating the object at different angles will shift different frequency components of the object's spectrum into the fixed microscope objective lens, enlarging the accessible object frequency domain. For conventional holographic diffraction tomography, the complex amplitude of the total field, $U(\mathbf{r}_T)$, is measured through interferometric or holographical approaches. As illustrated in Fig. S1a, an off-axis hologram contains the information about the total complex field, shifted by the oblique incident field in the Fourier domain. After converting the measured the total complex field $U(\mathbf{r}_T)$ to the first-order scattered field $U_{s1}(\mathbf{r}_T)$ according to the Born or Rytov

approximations [Eq. (S7)], the resultant 2D spectrum is projected onto the particular Ewald sphere in the 3D Fourier space, according to the relations given by Eq. (S6). By changing the incident angle of the illumination, the resultant first-order scattered field can access different regions of the 3D Fourier spectrum of the object, and eventually, a certain portion of $\hat{O}(\mathbf{u})$ can be retrieved, which will allow us the reconstruct the scattering potential of the 3D sample.

### 1.2 Transfer function theory for non-interferometric diffraction tomography

In this subsection, we establish transfer function theory for non-interferometric quantitative phase imaging (QPI) and diffraction tomography. Note that the 3D imaging formation under partially coherent illumination is analyzed in N. Streibl's work under the first-order Born approximation[7]. Here we extend it to the case of first-order Rytov approximation. With loss of generality, we first assume that the 2D or 3D sample is illuminated by a quasi-monochromatic plane wave with unit amplitude and imaged with a finite-aperture imaging system. Based on Eq. (S8) and Eq. (2) of the main text, the complex phase function of the first-order scattered field under the first-order Born or Rytov approximation can be expressed as

$$\begin{aligned}\phi_s(\mathbf{r}) &= \frac{1}{U_{in}(\mathbf{r})}U_{s1}(\mathbf{r}) \\ &= \frac{1}{U_{in}(\mathbf{r})}[O(\mathbf{r})U_{in}(\mathbf{r})]\otimes g(\mathbf{r}) \\ &= \frac{1}{U_{in}(\mathbf{r})}[a(\mathbf{r})U_{in}(\mathbf{r})]\otimes g(\mathbf{r}) + \frac{j}{U_{in}(\mathbf{r})}[\phi(\mathbf{r})U_{in}(\mathbf{r})]\otimes g(\mathbf{r})\end{aligned} \tag{S9}$$

where $O(\mathbf{r})$ is the 2D or 3D object function with its real (absorption) component $a(\mathbf{r})$ and imaginary (phase) component $\phi(\mathbf{r})$; $g(\mathbf{r})$ represents the complex point spread function (PSF) of the imaging system. According to Eq. (3) of the main text, the logarithmic value of the measured intensity image (or intensity stack for the 3D case) can be represented as

$$\begin{aligned}\ln I(\mathbf{r}) &= 2\mathrm{Re}[\phi_s(\mathbf{r})] \\ &= 2\mathrm{Re}[\frac{1}{U_{in}(\mathbf{r})}[a(\mathbf{r})U_{in}(\mathbf{r})]\otimes g(\mathbf{r}) + \frac{j}{U_{in}(\mathbf{r})}[\phi(\mathbf{r})U_{in}(\mathbf{r})]\otimes g(\mathbf{r})] \\ &= 2\mathrm{Re}[a(\mathbf{r})\otimes g'(\mathbf{r})] + 2\mathrm{Re}[j\phi(\mathbf{r})\otimes g'(\mathbf{r})] \\ &= a(\mathbf{r})\otimes 2\mathrm{Re}[g'(\mathbf{r})] + \phi(\mathbf{r})\otimes 2\mathrm{Re}[jg'(\mathbf{r})] \\ &= a(\mathbf{r})\otimes[g'(\mathbf{r}) + g'^*(\mathbf{r})] + \phi(\mathbf{r})\otimes[jg'(\mathbf{r}) - jg'^*(\mathbf{r})] \\ &= a(\mathbf{r})\otimes h_a(\mathbf{r}) + \phi(\mathbf{r})\otimes h_\phi(\mathbf{r})\end{aligned} \tag{S10}$$

where $g'(\mathbf{r})$ represents the PSF modulated by the incident field $U_{in}$; $h_a(\mathbf{r})$ and $h_\phi(\mathbf{r})$ represent the PSFs corresponding to the real (absorption) and imaginary (phase) components, respectively. Taking Fourier transform on both sides of Eq. (S9), we can get the logarithmic intensity spectrum

$$\ln\hat{I}(\mathbf{u}) = \hat{a}(\mathbf{u})H_a(\mathbf{u}) + \hat{\phi}(\mathbf{u})H_\phi(\mathbf{u}) \tag{S11}$$

where $\ln\hat{I}(\mathbf{u})$, $\hat{a}(\mathbf{u})$, and $\hat{\phi}(\mathbf{u})$ are the Fourier spectra of $\ln I(\mathbf{r})$, $a(\mathbf{r})$, and $\phi(\mathbf{r})$, respectively. $H_a(\mathbf{u})$ and $H_\phi(\mathbf{u})$ are the transfer functions for absorption and phase components, respectively. Under the coherent

illumination, $H_a(\mathbf{u})$ and $H_\phi(\mathbf{u})$ can be represented as[5]

$$\begin{aligned} H_a(\mathbf{u}) &= P(\mathbf{u}+\mathbf{u}_{in}) + P^*(\mathbf{u}-\mathbf{u}_{in}) \\ H_\phi(\mathbf{u}) &= P(\mathbf{u}+\mathbf{u}_{in}) - P^*(\mathbf{u}-\mathbf{u}_{in}) \end{aligned} \tag{S12}$$

where $P(\mathbf{u}+\mathbf{u}_{in})$ is the Fourier transforms of $g'(\mathbf{r})$, in which the shifts property of the Fourier transform is employed. $P(\mathbf{u})$ and $P^*(\mathbf{u})$ are the conjugated pair of the aperture function, where $P(\mathbf{u})$ represents the 2D complex pupil or 3D generalized aperture function of the imaging system[8]. Note that for diffraction tomographic imaging of 3D samples, the factor $4\pi j u_z$ of Eq. (S6) should be incorporated into the the 3D generalized aperture[5,9].

For a conventional microscope with the Köhler illumination configuration, the spatial coherence of illumination needs to be explicitly taken into consideration. When the illumination is produced by an incoherent extended source in the condenser aperture plane, the partially coherent image can be regarded as an incoherent superposition of coherent sub-images arising from all points of the incoherent source[5,10]:

$$H_\phi(\mathbf{u}) = \iint \left[P(\mathbf{u}+\mathbf{u}_{in}) - P^*(\mathbf{u}-\mathbf{u}_{in})\right] S\left(\mathbf{u}_{in_T}\right) d^2\mathbf{u}_{in} \tag{S13}$$

where $S$ is the intensity distribution of the incoherent source, $\mathbf{u}_{in} = (\mathbf{u}_{in_T}, u_{in_z})$ is the illumination wave-vector of incident field. For partially coherent illumination with circular or annular sources, the distribution of the 3D transfer functions can be calculated through analytical or numerical integration[7,9,11]. The 3D transfer function theory forms the basis of the 3D transport of intensity diffraction tomography (TIDT), which is based on non-interferometric, intensity only measurement akin to the 2D transport of intensity equation (TIE)[5]. By capturing the through-focus 3D intensity images, the scattering potential can be recovered by the 3D deconvolution based on the 3D transfer functions under the corresponding illumination condition[5]. The basic principle of non-interferometric diffraction tomography based on transport of intensity is illustrated in Fig. S1b, in which an example of intensity stack acquired under partially coherent circular illumination and its corresponding 3D Fourier spectrum are presented. The shadow area indicates the partially overlapping region in the phase transfer function between the positive and negative Ewald spheres. The amplitude of the phase transfer function is significantly attenuated due to the spectral cancellation, resulting in low-quality reconstruction, especially for low-spatial frequencies.

When the source size is sufficiently small (the illumination is nearly spatially coherent), the partial coherent phase transfer function reduces to the coherent one [Eq. (S12)], which contains two back-to-back Ewald spherical caps intersecting at the origin. The arc of Ewald sphere is shifted by incident illumination $\mathbf{u}_{in}$ and limited by objective pupil function $P(\mathbf{u})$, as illustrated in Fig. S1c. It can be seen that the object information on the two Ewald spheres can be transferred with maximum contrast (the amplitude of the phase transfer function is always one) without any spectral overlapping and cancellation. As detailed in the main text, the proposed transport of intensity diffraction tomography with non-interferometric synthetic aperture (TIDT-NSA) approach is based on 3D intensity-only measurements at various illumination angles. TIDT-NSA synthesizes the Ewald spheres at different illumination angles in the Fourier space, and the object spectrum can be filled by the extracted generalized apertures, allowing for the reconstruction of the scattering potential of the 3D sample with both high coherent phase contrast and incoherent diffraction-limited imaging resolution.

## 1.3 Effect of source temporal and spatial coherence on transfer function

For an ideal coherent imaging system, the illumination source is usually treated as an ideal point source with single wavelength emission. Nevertheless, in a practical microscopic imaging system, the effect of source temporal coherence (source spectrum) and spatial coherence (source size) should be explicitly considered for more accurate and realistic characterization of the transfer function. The temporal coherence of a light source depends on its power spectral density (PSD), i.e., spectral distribution. In the previous subsection, the light source is assumed to be a quasi-monochromatic extended source with an intensity distribution of $S(\mathbf{u})$. In this subsection, we assume that the light source has a certain spectral bandwidth with a PSD of $S_\omega(\mathbf{u})$. Since different wavelengths are uncorrelated, the total intensity distribution of the source is the integral of the PSD over all wavelengths $S(\mathbf{u}) = \int S_\omega(\mathbf{u})\, d\omega$. Consequently, the transfer function can be obtained by the superposition of the power density produced by different wavelength components[5]

$$H_\phi(\mathbf{u}) = \iiint \left[P_\omega(\mathbf{u}+\mathbf{u}_{in}) - P^*_\omega(\mathbf{u}-\mathbf{u}_{in})\right] S_\omega(\mathbf{u}_{in_T})\, d^2\mathbf{u}_{in} d\omega \tag{S14}$$

This conclusion seems straightforward because it only needs to integrate over all different wavelengths. However, it should be stressed that the complex aperture function $P_\omega(\mathbf{u})$ here is a wavelength-dependent function. Therefore, polychromatic illuminations can be used to achieve broader spectral coverage by synthesizing Ewald spheres of different radii[12,13]. However, the wavelength-dependent sample phase or RI induced sample dispersion and chromatic aberration often complicate accurate phase and RI determination. Therefore, in our TIDT-NSA system, the sample is illuminated with narrow-band monochromatic LED illumination, and the temporal coherence is considered here not to achieve broader spectral coverage but to compensate for the slight wavelength dependence of the transfer function.

In Fig. S2a, we illustrate the transfer function synthesis of temporal coherence (polychromatic source) and spatial coherence (extended source). The phase transfer function distribution of an ideal monochromatic coherent source is shown in Fig. S2b, which contains two perfect conjugated caps of Ewald spheres. The spatially coherent polychromatic transfer functions shown in Fig. S2c reveal the spectral extension and blurring effect due to the finite illumination wavelengths. The inside and outside boundaries of the transfer function are determined by the maximum and minimum illumination wavelength, corresponding to the Ewald spheres of different radii. All wavelength-dependent spherical caps are combined to build the full support of the polychromatic transfer function. On the other hand, the spatial coherence also contributes to the spectral extension and blurring of the transfer function, arising from the contributions of all source points, as shown in Fig. S2d. As indicated as the solid line, the centroid of the transfer function is located on the Ewald cap that corresponds to the weighted averaged illumination vector generated by an extended source, which is analogous to the ideal reflected by the definition of the "generalized phase" for partially coherent fields[14].

As a result, the spatiotemporal coherence of the illumination has significant effects on the imaging performance of a diffraction tomography system. The phase transfer functions of different illuminations with different spatiotemporal coherence are compared in Fig. S2e. It can be found that compared to the temporal coherence (by using polychromatic illumination), spatial coherence modulation (by using high

NA illumination from an extended source) is more effective in terms of expanding the spectral coverage of the transfer function, which leads to higher imaging resolution and better depth sectioning. With the increase in illumination aperture, the axial missing-cone problem is gradually alleviated, and the lateral and axial resolution is improved. However, the cancellation of phase transfer function contribution in the overlapping region (indicated by the gray shadow) becomes more prominent. Within the proposed TIDT-NSA technique, the full donut-shaped incoherent-diffraction-limited phase transfer function support can be intactly filled by the noninterferometic synthetic aperture of multi-angle coherent illuminations. The sequential synthetic aperture procedure allows the transmission of all spatial frequencies within the support without any overlapping, attenuation, or cancellation, endowing TIDT-NSA with capabilities of high signal-to-noise ratio and high-sensitivity RI detection.

## 2 Kramers-Kronig relations in diffraction tomography

### 2.1 Basic principle of Kramers-Kronig relations

The Kramers-Kronig relations describe the mathematical connection of square-integrable function with causality between its real and imaginary parts, and these relations have been widely applied in various research fields since its derivation by Kronig[15] and Kramers[16]. More specifically, the real and imaginary parts of a square-integrable function $f(x)$ that is analytic in the upper half-plane of $x$ satisfies the following equation

$$\begin{aligned}\mathrm{Re}\left[f(x)\right] &= \frac{1}{\pi} p.v. \int_{-\infty}^{\infty} \frac{\mathrm{Im}\left[f(x')\right]}{x'-x} dx' \\ \mathrm{Im}\left[f(x)\right] &= -\frac{1}{\pi} p.v. \int_{-\infty}^{\infty} \frac{\mathrm{Re}\left[f(x')\right]}{x'-x} dx'\end{aligned} \tag{S15}$$

where $p.v.$ indicates Cauchy principal value, and the corresponding integral region is formed by removing the pole $x'$ of the integral function by a very small semicircle. That is the entire upper half complex plane (with real axis) except for the pole of $x'$ in complex coordinate.

Recently, the Kramers–Kronig relations were introduced to the fields of QPI and ODT by Baek and Park[17]. They reinterpret the asymmetric-illumination-based non-interferometric QPI from the perspective of space-domain Kramers-Kronig relations. Since the Kramers-Kronig relations describe the connection between the real and imaginary parts of a complex analytic function, if a complex field is analytic in the upper half-plane, Kramers-Kronig relations hold between the real and imaginary parts of the complex field, and the phase (imaginary) component can be recovered from the intensity (real) measurement of the complex field.

### 2.2 Connections between Kramers-Kronig relations and transfer function theory

Considering a sample illuminated by a quasi-monochromatic plane wave with unit amplitude, the sample contribution can be represented as a complex phase function [see also Eq. (1) of the main text]

$$\phi_s(\mathbf{r}) = \ln\left[U(\mathbf{r})/U_{in}(\mathbf{r})\right] = \ln\left[1 + U_s(\mathbf{r})/U_{in}(\mathbf{r})\right] \equiv a(\mathbf{r}) + j\phi(\mathbf{r}) \tag{S16}$$

where $a(\mathbf{r})$ and $\phi(\mathbf{r})$ represent the real (absorption) and imaginary (phase) parts of the sample. The intensity that can be directly measured is connected with the real part of the complex phase function

$$I(\mathbf{r}) = |A(\mathbf{r})|^2 = \exp\left[2\mathrm{Re}[\phi_\mathrm{s}(\mathbf{r})]\right] \tag{S17}$$

As derived in Supplementary Information 1.1, under either the first-order Born ($U_s \approx U_{s1} \ll U_{in}$) or Rytov approximation ($U_s \approx U_{s1} = U_{in}\phi_s$), the complex phase can be represented as a normalized version of the first-order scattered field [Eq. (S8)], which is repeated here for convenience

$$\phi_s(\mathbf{r}) = U_{s1}(\mathbf{r}) / U_{in}(\mathbf{r}) \tag{S18}$$

The Fourier shift property suggests that dividing by $U_{in}$ in the spatial domain is equivalent to a shift of the signal spectrum in the frequency domain[3]. Considering Eq. (2) of the main text, the Fourier spectrum of the complex phase function can be written as

$$\hat{\phi}_\mathrm{s}(\mathbf{u}) = \hat{O}(\mathbf{u})\, P(\mathbf{u} + \mathbf{u}_{in}) \tag{S19}$$

Note that for 3D imaging, the factor $4\pi j u_z$ should be incorporated into the generalized aperture function. Eq. (S19) suggests that the support of the Fourier spectrum of the complex phase function is determined by the single shifted pupil function (instead of two as in the intensity spectrum of Eq. (S11)). The Titschmarch theorem states that a square-integrable function vanishing for a half-plane of the Fourier space results in its analyticity in the half-plane of the spatial domain[18]. The asymmetric Fourier spectrum implies the analyticity in the upper half-plane due to the interchangeability of causality and analyticity for the Kramers–Kronig relations. For QPI of 2D thin specimens, it is plain to see that when the matched illumination condition is satisfied, i.e., $|\mathbf{u}_{in}| = NA_{obj}/\lambda$, the complex phase function is half-plane analytic. Consequently, the Kramers-Kronig relations can be applied to infer the phase component (imaginary) $\phi(\mathbf{r})$ from the intensity (real) measurement. The real part of complex phase function $\phi_s(\mathbf{r})$ is connected to the measurable intensity:

$$\mathrm{Re}[\phi_s(\mathbf{r})] = \frac{1}{2}\ln I(\mathbf{r}) \tag{S20}$$

It should be noted that, different from the single side-band Fourier spectrum of complex phase function [Eq. (S19)], the logarithmic intensity spectrum [Eq. (S11)] contains two conjugated "twin" pupil functions as a result of the loss of imaginary part (phase information). When the illumination angle is smaller than that limited by NA of the objective lens, i.e., $0 < |\mathbf{u}_{in}| < NA_{obj}/\lambda$, the two pupil functions overlaps and cancel each other out at low frequencies, as shown in Fig. 1d of the main text. The information within the pupil function can be totally transferred if and only if the illumination angle matches the NA of the objective lens, i.e., $|\mathbf{u}_{in}| = NA_{obj}/\lambda$. It should be mentioned that the Kramers–Kronig relations are essentially a special case of the Hilbert transform. It is also well-known that the Hilbert transform can be concisely expressed as a half-plane $\pm\pi/2$ phase filter in the Fourier domain[19]. Therefore, the imaginary part of the complex phase function can be simply retrieved via applying half-plane Fourier filtering on the logarithmic intensity spectrum (keep only one of the"twin" pupil functions and remove the other).

To sum up, the matched illumination condition can be regarded as an equivalent of the analyticity condition of the complex phase function. Nevertheless, the transfer function theory presented here gives a more intuitive and complete physical picture behind the Kramers-Kronig relations, which is not only valid when the matched illumination condition is satisfied, but can also be used for resolution and contrast analysis under arbitrary illumination conditions, including partially coherent illuminations.

### 2.3 Analyticity analysis of the complex phase function in the 3D spatial domain

For QPI of 2D thin specimens, the analyticity of the complex phase function in the half-plane of the 2D spatial domain depending on the incident angle of the illumination matches the cut-off angle allowed by the objective pupil function. However, the matched illumination condition is difficult to strictly fulfill in practice, especially for high-NA microscopic systems (e.g., with an oil-immersion objective lens).

The situation will be completely different if we extend the intensity measurement into the 3D space. Similar to the 2D case, the Fourier spectrum of the 3D complex phase function contains a single shifted generalized aperture (Ewald spherical cap) [Eq. (S19)]. Interestingly, the support of the Fourier spectrum of the complex phase function is always vanishes for a half-plane of the 3D Fourier space, regardless of the illumination angle:

$$\hat{\phi}_s\left(u_x u_{in_x} + u_y u_{in_y} + u_z u_{in_z} < 0\right) = 0, (u_x, u_y, u_z) \neq 0 \tag{S21}$$

where $\mathbf{u}_{in} = (u_{in_x}, u_{in_y}, u_{in_z})$ is the incident frequency vector.

Similarly, the logarithmic intensity spectrum [Eq. (S11)] also contains two conjugated generalized apertures shifted according to the incident illumination angle, as illustrated in Fig. 1e of the main text. The two generalized apertures in the logarithmic intensity spectrum are mirror-symmetrically shifted in 3D space, and they never cancel each other out (except for the origin, which corresponds to the trivial constant phase) under arbitrary-angled illuminations. These observations inspire us to extend the Titschmarch theorem to the 3D case, exploiting 3D space-domain Kramers-Kronig relations to the 3D intensity (stack) instead of 2D intensity (image) to circumvent the restriction imposed by the matched illumination condition. Consequently, the Kramers-Kronig relations can be applied to retrieve the phase component (imaginary) $\phi(\mathbf{r})$ from the intensity (real) measurement by simply taking 3D Hilbert transform (or 3D Fourier domain half-space filtering equivalently) on the measured intensity stack. The 3D Fourier domain half-space filter is expressed as:

$$H_{HT}(\mathbf{u}) = \begin{cases} -j & \prod > 0 \\ 0 & \prod = 0 \\ +j & \prod < 0 \end{cases} \tag{S22}$$

where the 3D Fourier space is split in half with the 3D plane, which is perpendicular to the incident frequency vector $\prod(\mathbf{u}) = u_x u_{in_x} + u_y u_{in_y} + u_z u_{in_z}$.

### 2.4 Comparison of different 2D QPI and 3D ODT techniques

To demonstrate the advantages of the proposed TIDT-NSA over the state-of-the-arts, we compared it with interferometric/non-interferometric phase imaging (both 2D and 3D) and diffraction tomographic

RI imaging methods in Table S1. It should be emphasized that when we talk about QPI, it is usually assumed that the measured samples are 2D (thin) objects, which can be represented as a 2D complex function composed of the absorption component and the phase component. However, the phase delay induced by the object is only the axial projection (accumulation) of its 3D RI distribution. This problem can be effectively overcome by diffraction tomographic techniques, which enables high-resolution real 3D (lateral + axial) imaging of the 3D sample by recovering its 3D RI distribution.

As a classic interferometric approach for QPI, digital holographic microscopy (DHM) can measure the phase delay introduced by the heterogeneous RI distribution within the specimen with a single off-axis hologram. By combining DHM with ODT, 3D RI tomographic imaging can be achieved based on a set of quantitative phase distributions obtained by rotating the object or changing the illumination directions. Holographical ODT is the most classic ODT technique, which is compatible with both Born and Rytov approximations. Typically, several hundreds of holograms are required to fill the object Fourier spectrum sufficiently[20,21].

Transport of intensity equation (TIE) is a deterministic and non-interferometric phase retrieval approach base on intensity propagation[5]. To solve TIE, the axial intensity derivative should be calculated, which typically requires 3 intensity images taken at axially displaced planes (generally 3 frames). TIE can be extended to partially coherent fields, and the phase definition and the image reconstruction algorithm should be generalized by explicitly taking the partial coherence into account[5,14]. Differential phase contrast (DPC) is also a non-interferometric QPI method that utilizes a pair of images under asymmetric illumination patterns[22]. Generally, four intensity images corresponding to asymmetric and complementary illumination are required for quantitative phase reconstruction. Fourier ptychographic microscopy (FPM) is a high spatial-bandwidth product iterative QPI method based on ptychographic phase retrieval and coherent synthetic aperture[23]. Since FPM is also based on asymmetric illumination, DPC and FPM share some common features, but the latter can surpass the incoherent diffraction limit of the imaging system at the expense of acquiring a considerable number of darkfield images. The transfer function analysis of DPC and FPM reveals that the low-frequency phase component can only be recovered when the incident angle of the illumination matches the cut-off angle allowed by the objective pupil function[4,24]. Thus, the QPI technique based on Kramers-Kronig relations can be viewed as a special case of DPC or FPM under the matched illumination condition. All these QPI approaches can be extended to 3D RI tomography by simply incorporating the ODT principle, e.g. diffraction tomography based on Kramers-Kronig relations demodulation (K-K DT)[17], which is similar to the conventional holographical ODT except that QPI is realized noninterferometically.

The intensity diffraction tomography (IDT)[25,26]and Fourier ptychographic diffraction tomography (FPDT)[3,27] are the 3D counterparts of DPC and FPM by extending them from 2D QPI to 3D non-interferometric diffraction tomography. They are based on a similar optical setup, but FPDT can achieve high spatial-bandwidth product RI imaging by incorporating darkfield intensity measurements. Note that the matched illumination condition is also required for accurate reconstruction of low-frequency RI for both IDT and FPDT. Phase retrieval instrument with super-resolution microscopy (PRISM) is based on a noninterferometic multi-plane phase retrieval approach under partially spatial, and partially temporal

(polychromatic) illumination[13]. It can be viewed as an extension of white-light diffraction tomography (WDT) approach[28] to partially spatial coherent illumination. Although the matched illumination condition is not required in PRISM, this method can only be classified as 3D QPI rather than ODT because only the phase stack of the 3D sample can be recovered.

Different from the above state-of-the-arts, TIDT-NSA combines through-focus scanning with the illumination angle diversity. Compared with traditional ODT techniques, TIDT-NSA does not require coherent illumination and interferometric measurement. The z-scanning eliminates the need for the matched illumination condition, and the sequential synthetic aperture procedure allows for high signal-to-noise ratio and high-sensitivity RI detection at incoherent diffraction-limited imaging resolution.

## 3 Hardware implementation for TIDT-NSA

### 3.1 Optical platform for TIDT-NSA

To conduct both configurations at the front-end illumination and back-end acquisition, we built TIDT-NSA platform based on a commercial microscope (IX83, Olympus) assisted with angle-varied LED illumination and motorized focus drive scanning mechanism. The illumination set consisting five LED rings (Adafruit) with different diameter sizes are stitched together as the source, and the LED set is placed 25 mm away from the sample to provide illuminations from different angles with the maximum illumination NA of 0.95 ($\sim$ 72 degrees, red arrow in Fig. S3b), whose center is aligned with the optical axis of imaging system. Each LED (WS2812B, SMD-5050) approximately consumes 200 mW of power and provides spatially coherent quasi-monochromatic illumination for three individual channels (central wavelength Red 629 nm, Green 520 nm, Blue 483 nm, and $\sim$20 nm bandwidth). The photos of multi-annular programmable LED source and single LED device are both illustrated in Fig. S3a. The illuminating beam of each LED element is controlled to turn on sequentially by an ARM board controller (MIMXRT1062, ARM Cortex-M7 @Core clock 600 MHz, Teensy 4.1, NXP Semiconductors) and it passes through the sample with arbitrary tilted angles. An sCMOS camera with 82% peak quantum efficiency (ORCA Flash 4.0 V3 C13440, Hamamatsu, pixel resolution 2048 $\times$ 2048, pixel pitch 6.5 $\mu$m) is employed for 3D intensity stacks recording under detection objective (40$\times$/0.95 UPlanSApo, Olympus) for unstained cell (including MCF-7, RAW 264.7, C2C12, *C. elegans*, and HeLa cells) under red LED illumination channel and another objective (100$\times$/1.4 Oil, UPlanSApo, Olympus) for samples of polystyrene beads and HepG2 cells blue LED illumination channel. Software in data acquisition computer (Intel Core i7-7820X processor operating at 3.6 GHz with eight cores and 16 threads, 128 GB of 2133 MHz DDR4 RAM, and NVIDIA GeForce RTX 2080Ti 11GB) communicates with the camera and focus stage drive via the $\mu$Manager-API and Java programs. The camera is synchronized with the LED array by the controller via two coaxial cables that provide the trigger and monitor the exposure status, and ARM controller board delivers a series of deterministic transistor–transistor logic (TTL) triggers for camera frame-grabber. As illustrated in Fig. S3c, the triggers including the control of motorized focus drive positioning, LED illumination data transmission and blanking, and external camera fire and frame grabber are both tightly synchronized with each other through the hardware trigger single.

### 3.2 Electromechanical system synchronization

We designed a synchronization paradigm which efficiently coordinated the LED illumination pattern switch, focus stage movement, and camera readout interval. For example, in the process of TIDT-NSA cycle, a set of 128 raw intensity stacks containing 201 axial displaced frames under different illumination angles are captured to reconstruct a RI volume image. The imaging protocol can be described in detail as follows: the first and the last intensity stack of each stacks group are set as the start and end markers located on the $1^{st}$ and $5^{th}$ LED rings to provide the maximum and minimal NA of illumination, respectively. Under a certain illumination angle, the high-precision focus-drive is controlled to scan the different focal planes by the $\mu$Manager software, and this software is synchronized to the ARM board through USB slave device mode for the transfer of drive signal and step done flag. To minimize the exposure time, we use delicate time sequence to synchronize the movement of focus drive and the exposure of the sCMOS camera, and the time sequences for the camera acquisition are shown in the top inset of Fig. S4. Moreover, we use the external trigger mode of sCMOS camera together with ARM board to control the LED illumination switching synchronously during exposures of rows. Due to the accurate synchronization between the sequence of each intensity stack exposure and angled-LED illumination, this acquisition mode is equivalent to the use of a global-shutter mode. Moreover, to minimize the vibrational noise and time-dependent motion artifacts, we reduce the exposure time to 50 ms, as shown by the focus drive state signal in the lower inset in Fig. S4. Notably, while the sCMOS camera is working in the external trigger mode, we delay a short time before the intensity stacks acquisition of new frame group data in order to avoid variations in actual exposure time and unstable initial state of focus scanning stage.

To eliminate the high-order residue background in reconstruction and homogenize the illumination amplitude, we capture a series of extra group of background intensity stacks under each illumination angle with only the coverslip under the microscope objecitve. For the acquisition of fixed sample, total 25728 intensity frames are acquired within a 4D stack ($2048\times2048\times201\times128$) for 201 axial slices and 128 LED illuminations under $40\times$ 0.95 NA objective, and the whole cycle of TIDT-NSA requires over 1.5 hour acquisition time (200 ms exposure time for each frame). While for dynamic HeLa time lapses imaging, we adjust the resistance value of current output limiting resistor to provide sufficient total photon flux within 50 ms of exposure time. Besides, the number of $z$-step slices at different planes and illuminations are both down-sampled to achieve the speed limit of system, and the 4D intensity stack contains 204 intensity frames ($2048\times2048\times17\times12$) for each 3D stack are captured within 20 secs (50 ms for each frame). We implement the proposed TIDT-NSA reconstruction algorithm on a workstation computer with 128 GB DDR4 RAM, and the time-lapse intensity stack and background stack are parallel feeded into the program as TIFF-stack files. The result is also saved as TIFF-stack files and can be easily analyzed using ImageJ. The $\mu$Manager-API, Java programs, C++ Microsoft Foundation Classes (MFC) framework, and C language programs are used for software and hardware data I/O and efficient computation during image acquisition and reconstruction.

# 4 TIDT-NSA reconstruction algorithm

Following the pipeline of TIDT-NSA reconstruction algorithm in Fig. S5, the detailed working flow can be schematically described as follows: 1) We calibrate the illumination angles, which results in the shift of Ewald sphere spectrum, and the background intensity stacks under each LED illuminations without samples are taken for the background artifact removing initially. 2) The intensity stacks of object are captured under the precise synchronization of an imaging system, and 3D Fourier transform is implemented on the background normalized stacks. By applying 3D half-space Fourier filtering (space-domain Kramers–Kronig relations equivalently) on each logarithmic 3D intensity spectrum, the corresponding 3D phase distributions (the imaginary part of the complex phase function) under different incident illuminations can be retrieved. 3) 3D deconvolution is further performed on the preliminary synthesized spectrum based on the incoherent transfer function, and 3D RI tomographic reconstruction can be realized finally.

### Step 1: Illumination angle calibration and intensity background normalization

For the LED illumination calibration process, we reorder 128 intensity images of pure phase object on the in-focus plane, and the numerical self-calibration procedure of LED rings are performed in the frequency domain[29]. The algorithm imposed here follows two geometric constraints. First, the distribution of our LED ring set is expected to obey multi-concentric circular geometry. Second, the LEDs on each ring arrangement board are expected to be equally spaced. Correspondingly, each ring of LEDs contains $2\pi$ radian angular space, and each pair of neighboring LEDs occupies a $2\pi/N$ radian, where $N$ is the LED number on each ring. Our LED position calibration algorithm starts with an initial guess (blue star in Step1 of Fig. S5), and the initial estimated LED positions are often contaminated by noise. Accordingly, the final calibrated LED positions are parameterized as a nonlinear fitting between the raw calibration results and circular geometry constraint of LED ring set. By solving the optimization problem, the LED portion can be accurately calibrated and the calibrated results are shown in Step 1 of Fig. S5.

As to the background normalization process, 128 raw intensity stacks of object $I_B$ under tomographic illumination angles identical to the background data acquisition $I_{Raw}$ are recorded within the same exposure time, and the background processed object intensity stacks are obtained by the normalization between raw intensity stack and background intensity distribution $I(\mathbf{r}) = I_{Raw}(\mathbf{r})/I_B(\mathbf{r})$. Note that the background normalization is a mandatory step to compensate the illumination inhomogeneities and ensure that the normalized illuminations from all LED elements are uniform and of unit amplitude.

### Step 2: Image stacks acquisition and spectrum separation in Fourier space

After background normalization, the 3D logarithmic intensity spectrum could be calculated by taking 3D Fourier transform on each processed intensity stack, $\ln\hat{I}(\mathbf{u})$. Then, the corresponding 3D phase distributions (the imaginary part of the complex phase function), $\phi(\mathbf{r})$, under different incident illuminations can be obtained by applying 3D half-space Fourier filtering based on [Eq. (S22)], which is equivalent to applying the 3D space-domain Kramers-Kronig relations to each 3D intensity stack (see Step 2 in Fig. S5).

### Step 3: Synthetic aperture and scattering potential reconstruction

After implementing the 3D half-space Fourier filtering on each dual sideband 3D spectrum, the resultant spectra of 3D scattered field both containing real and imaginary parts of complex phase function are synthesized together in the Fourier space to get a preliminary estimate of the 3D object spectrum $\hat{O}_{syn}(\mathbf{u})$ (see Step 3 in Fig. S5). To further compensate the effects of LED elements discretization and the partially coherence (both temporal and spatial) of the illumination, a simple regularized 3D deconvolution is further performed on the preliminary synthesized spectrum based on a synthesized transfer function taking the LED discrete sampling, partial coherence of the illumination, and the correction factor [the $4\pi ju_z$ factor in Eq. (S5)] into accounts. The synthesized transfer function is calculated by numerical integration according to Eq. (S14), as discussed in Subsection 1.3 of the Supplementary Information. The regularized 3D deconvolution can be represented as

$$\hat{O}(\mathbf{u}) = \frac{\hat{O}_{syn}(\mathbf{u})H^*_{syn}(\mathbf{u})}{H_{syn}(\mathbf{u})H^*_{syn}(\mathbf{u}) + \varepsilon} \tag{S23}$$

where $\hat{O}$ and $\hat{O}_{syn}$ are the finally deconvolved spectrum of object scattering potential and preliminary synthesized spectrum, respectively. $H_{syn}$ is the synthesized 3D transfer function of the system, and $\varepsilon$ is a small regularization parameter to prevent the over-amplification of noise. It should be noted that, when the illumination angles are sufficiently diverse and uniformly distributed within the objective pupil, the synthesized 3D transfer function $H_{syn}$ should approach an ideal 3D incoherent optical transfer function (OTF) of a conventional incoherent microscope. Different from the conventional 3D incoherent OTF where the high spatial frequencies are strongly attenuated, the sequential synthetic aperture procedure in TIDT-NSA allows the transmission of all spatial frequencies within the support without any attenuation. After deconvolution, a hybrid iterative constraint algorithm combining non-negative constraint and total variation regularization is further applied to computationally fill the missing cone information. Finally, the scattering potential of the object is reconstructed after 3D inverse Fourier transform, and the corresponding 3D volumetric RI distribution can be used for the label-free 3D imaging of biological samples, as shown in the Step 3 of Fig. S5.

# 5 Validation of TIDT-NSA on both simulations and experiments

## 5.1 Simulations on 2D numerical propagation and 3D scattering

We first simulate a pure phase sphere as the test sample, and the optical parameters are the same with the bead experiments in the main text. The bead diameter is 5 $\mu$m with pixel sampling rate of 0.065 $\mu$m in lateral direction and 0.65 $\mu$m in axial direction. Figures S6a and b illustrate the 2D spectra projection of 3D complex phase function of an ideal phase bead $\phi_s$ on in-focus plane from two different illumination directions, respectively, and the real and imaginary parts of 2D complex total field $U = U_{in}\exp(\phi_s)$ can be obtained by implementing inverse Fourier transform. By further invoking the numerical propagation of 2D complex field under the corresponding illumination angle, the resultant 3D complex field of object both containing intensity and phase stacks are acquired, as shown in Figs. S6c and d. Because the intensity information can only be captured in bright-field imaging system, and the phase component in complex

amplitude is lost as a result. As illustrated in Figs. S6e and f, the propagated 3D intensity stack corresponds the actually captured intensity stack in an actual imaging system, and the distribution of 3D intensity spectrum still exhibits dual side-band features of Ewald sphere arcs. Also, we can observe the spectrum leakage both in the logarithmic intensity spectrum of Figs. S6e and f, and the reason for this phenomenon is the energy leakage in the propagation of 2D complex amplitude along z-axis induced by the unsatisfied boundary conditions for Fourier transform. By performing 3D half-plane Fourier filtering on each dual side-band 3D spectrum, analyticity interpretation of 3D Kramers–Kronig relations in frequency space equivalently, the preliminary estimate of the 3D object function can be obtained by the process of synthetic aperture of each resultant spectra of 3D scattered field. The purpose of simulations on 3D scattering and 2D numerical propagation is to reveal the reversible relations between the forward and inverse model of 2D complex phase function and 3D complex scattering field retrieval.

### 5.2 Experimental results on polystyrene beads

Additionally, we provide more information about the experimental tomographic result of single phase bead and the comparison for the enhancement of low-frequency components within various non-interferometric tomography approaches in Fig. S7. Figure S7a illustrates the raw synthetic spectrum of bead, and the slight Gibbs rings distribution in the raw spectrum verify the validity and accuracy of the proposed synthetic aperture method. The iterative reconstruction algorithms with non-negative and total variation regularization are applied to the final RI stack for accurate 3D RI reconstructions, and the dense concentric ring pattern (sinc function) can be distinguished and resolved in the constrained lateral spectrum cross section (Fig. S7b, arrows). The iterative constraint algorithm generally generates reasonably accurate predictions for the missing angle region which cannot be covered by the direct deconvolution of transfer function. After iterations of constraint, the negative RI bias is removed and the missing cone effect is significantly alleviated. The final RI distributions of reconstructed micro bead with 3 $\mu$m diameter are presented in Fig. S7c with sampling rate of 0.065 $\mu$m in all directions. Due to the missing cone issue as well as the isotropic coverage of the Fourier spectrum in lateral and axial directions, the recovered bead still suffers from slight elongation and RI underestimation along the axial direction, as shown in Fig. S7d.

We implement 2D TIE phase reconstruction utilizing a set of three intensity images on the cluster of phase beads, and the recovered phase distribution of these beads along lateral and axial directions is illustrated in Fig. S7e. However, this phase distribution of bead cluster only represents the 3D coherent scattered field along axial direction and does not contain any optical sectioning information of object. Figures S7f-h illustrate the tomographic results of polystyrene beads utilizing intensity diffraction tomography (IDT), Fourier ptychographic diffraction tomography (FPDT), and TIDT-NSA under non-matched illumination condition (0.95 $NA_{ill}$ to 1.4 $NA_{obj}$). For the cases of IDT and FPDT, the low-frequency phase components (near zero frequency) in the 2D in-focus intensity images are overlapped with each other, and the low-frequency spectral overlapping region can never be recovered due to the violation of analyticity of corresponding complex phase function. While under the situation of TIDT-NSA, the two conjugated Ewald spheres hardly ever intersect except for the origin and the low frequency components can be totally recovered under arbitrary illumination conditions. Existed TIE-based phase retrieval is more suitable for quantitative phase reconstitution of 2D thin object rather than 3D object, and

the benchmark with the reconstruction from 2D TIE is essential to demonstrate the advance of 3D intensity transport of TIDT-NSA. Also, the tomographic RI results based on this monolayer cluster of beads are presented to verify the enhanced ability of 3D RI low frequency recovery of TIDT-NSA compared with other non-interferometric diffraction tomography. It could be observed from the experimental comparison results that the low-frequency phase component of sphere are almost lost for the approaches of IDT and FPDT, while TIDT-NSA is more beneficial to the recovery of low-frequency, demonstrating the advantages of proposed TIDT-NSA in low-frequency enhancement.

### 5.3 Benchmarking of TIDT-NSA against with other diffraction tomographic methods

In order to verify the performance of TIDT-NSA among other diffraction tomographic imaging approaches, we proposed the benchmarking results of TIDT-NSA against other 3D phase imaging methods (IDT and FPDT). Figure S8 shows the comparison results on unicellular diatom microalgae fixed in glycerin gelatin imaged with both 0.95 $NA_{ill}$ and $NA_{obj}$ (matched illumination condition), and the whole view of both 3D RI and absorption measurements are illustrated in Figs. S8a and b, respectively. Moreover, an example region of interest image at two different planes is shown in Figs. S8c and d. The "phase" features of pores and spines are visible in both three diffraction tomographic methods (Z plane1), but the RI distribution of proposed TIDT-NSA is sharper and more vivid than the distribution of IDT and FPDT (indicated by the white circles) and the structure of ridge and context with relatively high absorption are clear and distinguishable. Moreover, both the lateral resolution and contrast are preserved in TIDT-NSA while recovered absorption diatom structure is lost in IDT and FPDT at large defocus from the results of RI and absorption on another farther plane (Z plane2). Line profiles in Figs. S8e and f provide intuitive quantification of 3D tomographic reconstruction at different planes. Despite the lack of ground truth of these 3D biological objects, which is challenging to measure experimentally, the reconstructed comparison results shown in Fig. S8 demonstrates the tomographic quality improvement and difference (significantly for large defocusing plane) over the existed method back-to-back.

## 6 Quantification and analysis of TIDT-NSA

### 6.1 Resolution characterization with USAF target and nano-sphere

We use the UASF and star target to benchmark the resolution limit of TIDT-NSA, and the imaging resolution analysis and characterization are provided as well. By invoking Abbe's diffraction limit criterion, the imaging resolution for a coherent imaging system is given by $\Delta d \cong \frac{\lambda}{n \sin \alpha} = \frac{\lambda}{NA}$, where $\lambda$ is the illumination wavelength and NA is the optical system numerical aperture.

The Abbe limit represents the highest spatial frequency which is corresponding to the half width of incoherent diffraction limitation for bright-field measurements. In this work, more commonly used Nyquist–Shannon sampling limit is utilized for full period limit of resolution characterization. In the diffraction tomography imaging system, the oblique illumination extends the transverse spatial frequency limit, as demonstrated by the two mirrored Ewald spheres along with the maximum NA of illumination in Fig. S1. In theory, the scattered field essentially poses a frequency shift in the detection frequency domain,

which extends the bandwidth in the transverse frequency domain by the following equation:

$$\mathbf{u}_{syn} = \mathbf{u}_{obj} + \mathbf{u}_{in} \tag{S24}$$

where $\mathbf{u}_{obj}$ and $\mathbf{u}_{in}$ indicate the transverse frequency projections of the maximum NA of the detection objective and illumination spatial frequency, respectively. As a result, the effective numerical aperture of the TIDT-NSA system can be given as

$$NA_{syn} = NA_{obj} + NA_{ill} \tag{S25}$$

We use dry and another oil-immersion objectives for different experiments, and the source unit of LED ring set is fixed to provide $NA = 0.95$ illumination. Respectively, the red LED illumination with 629 nm wavelength is employed for dry objective lens experiments, while the blue channel with 483 nm illumination is used in oil-immersion experiments. Note that the maximum NA detected by the objective is also limited by the RI of immerse medium, and there are different immerse mediums used in experiments as well. For example, the live cells are maintained in culture medium ($n_m$ = 1.33), while the polystyrene bead is immersed in Cargille matching oil ($n_m$ = 1.58). When we use 0.95 $NA$ with both of detection and illumination ($\lambda$ = 629 nm), the theoretical transverse resolution of TIDT-NSA system is about 330 nm, which is far beyond than the minimum bar in USAF phase target (Minimum 10-6, 548 nm full period width). The related resolution benchmark experiments are illustrated in Fig. S9a-d on phase resolution target both on USAF pattern and Siemens star pattern. Due to the missing of low-frequency components partially induced by missing cone issue, the phase values inside the patterns is missing but high-frequency features of retrieved phase pattern are still preserved, and the post-process constraint algorithm might alleviate this artifact.

For the case of oil-immersion objective under $NA_{ill} = 0.95$ LED illumination @483 nm wavelength, the resolution target is immersed in objective matching oil ($n_m$ = 1.518), thus the theoretical lateral resolution $\lambda/(NA_{ill} + NA_{obj})$ is about 206 nm. The absorption USAF resolution target elements with line pitch of 137 nm are chosen as the observed region (Minimum bar 11-6, 274 nm full period width), as illustrated in Figs. S9e-g. The axial resolution of TIDT-NSA system is complicated by the nonuniform axial distribution of Fourier spectrum in the spatial frequency (Fig. S1b, right panel). The missing cone issue near the low zero frequency area results in the prolongation of system PSF in the longitudinal direction. As for the axial resolution of diffraction tomography, the maximum resolution is given by the theory of $\lambda/(n_m - \sqrt{n_m^2 - NA_{obj}^2})$, and the full width at half maxima of line profiles across the single layer of USAF target in Fig. S9g demonstrate the near diffraction-limited axial resolution of 0.52 $\mu$m, confirming a depth resolution within the 0.55 $\mu$m range (in accordance with the theoretical prediction 0.52 $\mu$m). The double-headed arrows indicate the measured full-width at half-maximum of monolayer of USAF target akin to the PSF in the axial direction.

Besides, we use 100 nm diameter nano-sphere, satisfying sub-diffraction sized object, to demonstrate the ability of 3D RI tomography and characterize the achievable imaging resolution of proposed method. The beads are embedded in RI heterogeneous matching oil $n_m$ = 1.58. As shown in Fig. S9h, the experimental and simulated PSFs indicate the recovered RI distribution of sub-diffraction sized pure phase

bead. Axial and lateral line profiles across the phase PSF are also plotted in Fig. S9i. The RI distributions of experimental and simulated results are basically consistent with each other, supporting the claimed resolution of ∼206 nm laterally and ∼520 nm axially. Overall, the experimental results based on USAF target and nano-sphere for the characterization of resolution are sufficient to demonstrate the feasibility and capability of the proposed TIDT-NSA for non-interferometric, accurate 3D RI reconstruction from intensity-only measurements.

### 6.2 Analysis on the measurement accuracy and sensitivity of TIDT-NSA

Although the experimentally recovered volumes exhibit RI ranges matching expected biological values, the inherent variability of these specimens prevents quantitative analysis of the system's accuracy and sensitivity for recovering the true RI distribution and detecting small RI variations, respectively. Similar to the resolution benchmarking of RI, we further evaluate the modality's accuracy and sensitivity. Following the recent works about sensitivity analysis[30,31], it is possible to explore such experimental sensitivity analyses in quantitative phase systems with rigorous testing using expensive hardware which is not readily available for the TIDT-NSA system. Due to the lack of manufactured and well-characterized structures, thus, determining TIDT-NSA's accuracy and sensitivity is a challenging task. Here, we instead evaluate TIDT-NSA in simulation to determine its theoretical accuracy and sensitivity over the RI range present in our experimental data. Our simulations are performed with three primary components: 1) a ground-truth object, 2) a rigorous forward model generating intensity stacks under different illumination angles, and 3) our proposed TIDT-NSA reconstruction algorithm.

For the simulated object, we generate 3×3 sphere arrays with variable RI inside a volume of $33 \times 33 \times 33\ \mu m^3$, and each phase sphere is spatially separated in lateral and axial dimensions. (Fig. S10a). For the RI of phase bead, we assume a homogeneous imaging medium ($n_m$ = 1.58) and generate arrays with RI range Δn = [0.01, 0.02, 0.03, 0.04, 0.05] following the equation RI = $n_m$ + Δn. Other optical parameters are consistent with the actual experiments, and these parameters allow the evaluation of both TIDT-NSA's accuracy and its sensitivity to small RI changes across a large contrast range. According to the simulation approach of forward model in subsection 5.2, we propagate the final field through a 0.95 NA, 40× objective lens under different LED illumination angles and generate 128 intensity image stacks of the simulated sphere array. Furthermore, we added white Gaussian noise to the intensity images to generate SNR ranging from 1 to 10 and 20 realizations for each SNR level, as shown in Fig. S10b. The SNR is quantified by the ratio between the signal contrast and noise level as SNR = $\delta_{Signal}/\delta_{Noise}$, where $\delta$ denotes the standard deviation. This forward model with noise addition efficiently simulates 3D scattering of object, making it ideal for evaluating TIDT-NSA's recovery capabilities.

We repeat this simulation process on sphere array with differing RI and reconstruct the object volume using the proposed TIDT-NSA algorithm. We then compare the median recovered RI over the sphere area with the ground truth object to determine the reconstruction accuracy. For the reconstruction sensitivity, we evaluate the separation between the small RI variations Δn from the central RI value $n_m$. The simulation results for accuracy and sensitivity are summarized in Fig. S10c, and the left panel of Fig. S10c shows the average RI mismatch between our reconstruction and the ground truth across RI over the 20 realizations simulated under SNR matching our experimental condition. The error bars show the standard deviation in

this mismatch over these realizations, and the underestimations for large RI contrast objects are expected due to the presence of multiple-scattering invalidating the scattering assumption of the TIDT-NSA model. Right panel of Fig. S10c shows theoretical sensitivity range across multiple SNR conditions for different cases of RI contrast Δn. These results indicate that TIDT-NSA exhibits high sensitivity to RI variations across the full reconstructed volume under low-contrast imaging conditions. Our simulation shows that TIDT-NSA provides high-accuracy and high-sensitivity RI recovery of volumetric biological samples under the proper conditions. Given the assumption of more weakly scattering samples within our model's validity range, TIDT-NSA can recover correct accurate RI values and detect small fluctuations to variations in the object's RI. This analysis is promising for biological sample evaluation where these small RI variations is corresponding to the presence of pathogens in cells[32]. Although the evaluation of accuracy and sensitivity will suffer from experimental factors, including objective aberrations and illumination misalignments, our simulations shown here indicate TIDT-NSA provides accurate, highly sensitive volumetric recoveries of biological samples.

Moreover, we intend to provide the quantitative comparison about the reconstructed RI accuracy between the cases of 128 LED elements for fixed samples and 12 LED elements for live samples (10-fold less illumination number). Figures S10d and e illustrate the recovered Fourier spectrums and 3D RI slices of a cluster of polystyrene beads. It can be found that the sparse sampling of spectrum results in the slight degradation in RI value of 3D beads, and two line profiles are plotted to illustrate the RI value distribution of beads, as illustrated in Figs. S10f and g. In summary, the full LED illumination angles corresponding to the denser sampling in frequency spectrum enhance the value and accuracy of RI distribution. Even though the missing of low frequency effects the reconstruction accuracy, the issues of frequency missing can be relieved through the post-processing algorithm.

# 7 Supplementary experimental results of biological cells

## 7.1 Label-free 3D RI tomography on fixed cell clusters

Figure S11 illustrates the RI tomography on fixed cell clusters of murine skeletal myoblasts C2C12 cells. The RI change at different axial planes and the views in three orientations simultaneously at different lateral positions show the ability of 3D RI depth sectioning of TIDT-NSA. Also, four regions of the reconstructed C2C12 cells RI in Fig. S11 are expanded, highlighting our depth sectioning reconstructions. Figure S12a shows the reconstructed central RI slice of unstained RAW 264.7 cells using a 40× objective with a full field of view (FOV) of (333 × 333 $\mu$m$^2$), and three representative regions of interests, labeled as ROI1, ROI2, and ROI3, are further enlarged in Fig. S12b for better demonstration of cell morphology details at different axial planes. The cells are attached to the coverslip substrate, and fiber filopodial structures are observed at different depths due to the small tilt of the sample plane. In addition, other subcellular features, like the high-RI nucleus and some parts of the cytoplasm, are distributed over a limited depth range of ∼6 $\mu$m. Figure S12c and Supplementary Video 4 show two volume-rendered 3D RI distributions of one single cell from ROI3 and the small cell cluster from ROI2, in which the 3D structures of cytoplasm, cytoskeleton, microfilaments, and their interconnections are clearly resolved and comprehensively revealed. The unstained HepG2 liver carcinoma cells are imaged with a 100× objective,

and the reconstructed central RI slice is presented in Fig. S12d. From the two ROIs of the tomogram, cell membrane wrinkle, cell boundaries, and other high-RI cellular organelles, including the nucleoli, nucleus border cytoskeleton, and lipid droplets, are highlighted at different axial positions (Fig. S12e, arrows and circles). As illustrated in Fig. S12f, the line profiles across two cells at two different axial planes in Fig. S12e indicate that the RI distribution inside the nucleus is highly inhomogeneous.

### 7.2 Time-lapse tomographic imaging of apoptotic process of HeLa cell

For the dynamic experiments of HeLa cell, the full frame intensity stacks are down-sampled to 12 intensity stacks (204 intensity images), and these intensity images are captured within 22 secs, to minimize the vibrational noise and time-dependent motion artifacts. Thus, this time interval is basically enough for the 3D imaging of live cell with negligible motion blur artifacts. Moreover, the power of a single LED illumination source used in experiments is about 200 mW and the max illumination angle is about 150 degrees. Therefore, the lower density of LED illumination with so large divergence angle is far smaller than laser source resulting in the negligible photodamage in the dynamic experiments. We implement the 3D imaging of live HeLa cells under dry objective with 0.95 NA, and the full donut-shaped phase transfer function region is filled by the coherent illumination synthetic aperture, which corresponds to the 3D incoherent diffraction limit. The claim of lateral and axial imaging resolution of HeLa cells is consistent with characterization of resolution in subsection 6.1, showing the same imaging resolution with control sample of incoherent diffraction limit in the frequency domain. Moreover, Figure S13 illustrates the dynamic 3D RI results over hour-long time-lapse containing cell movement in the apoptosis process. This non-interferometric 3D label-free technique enables the monitoring of structures and dynamics in cellular processes that are prone to phototoxicity over a prolonged period of time, and the dynamic tomographic results demonstrate the high volumetric and high resolution imaging capacity of the proposed TIDT-NSA.

## References


1. Wolf, E. Three-dimensional structure determination of semi-transparent objects from holographic data. *Opt. Commun.* **1**, 153–156 (1969).
2. Lauer, V. New approach to optical diffraction tomography yielding a vector equation of diffraction tomography and a novel tomographic microscope. *J. Microsc.* **205**, 165–176 (2002).
3. Zuo, C. *et al.* Wide-field high-resolution 3d microscopy with fourier ptychographic diffraction tomography. *Opt. Lasers Eng.* **128**, 106003 (2020).
4. Sun, J. *et al.* Single-shot quantitative phase microscopy based on color-multiplexed fourier ptychography. *Opt. Lett.* **43**, 3365–3368 (2018).
5. Zuo, C. *et al.* Transport of intensity equation: a tutorial. *Opt. Lasers Eng.* 106187 (2020).
6. Cotte, Y. *et al.* Marker-free phase nanoscopy. *Nat. Photonics* (2013).
7. Streibl, N. Three-dimensional imaging by a microscope. *J. Opt. Soc. Am. A* **2**, 121–127 (1985).
8. Noda, T., Kawata, S. & Minami, S. Three-dimensional phase contrast imaging by an annular illumination microscope. *Appl. Opt.* **29**, 3810–3815 (1990).
9. Bao, Y. & Gaylord, T. K. Quantitative phase imaging method based on an analytical nonparaxial partially coherent phase optical transfer function. *J. Opt. Soc. Am. A* **33**, 2125–2136 (2016).
10. Zuo, C. *et al.* High-resolution transport-of-intensity quantitative phase microscopy with annular illumination. *Sci. Rep.* **7**, 7654 (2017).
11. Li, J. *et al.* Three-dimensional tomographic microscopy technique with multi-frequency combination with partially coherent illuminations. *Biomed. Opt. Express* **9**, 2526–2542 (2018).
12. Kim, T. *et al.* White-light diffraction tomography of unlabelled live cells. *Nat. Photonics* **8**, 256–263 (2014).
13. Descloux, A. *et al.* Combined multi-plane phase retrieval and super-resolution optical fluctuation imaging for 4d cell microscopy. *Nat. Photonics* **12**, 165–172 (2018).
14. Zuo, C. *et al.* Transport of intensity phase retrieval and computational imaging for partially coherent fields: The phase space perspective. *Opt. Lasers Eng.* **71**, 20–32 (2015).
15. Kronig, R. d. L. On the theory of dispersion of x-rays. *J. Opt. Soc. Am. A* **12**, 547–557 (1926).
16. Kramers, H. A. La diffusion de la lumiere par les atomes. In *Atti Cong. Intern. Fisica (Transactions of Volta Centenary Congress) Como*, **2**, 545–557 (1927).
17. Baek, Y. & Park, Y. Intensity-based holographic imaging via space-domain kramers–kronig relations. *Nat. Photonics* **15**, 354–360 (2021).
18. Titchmarsh, E. C. *Introduction to the theory of Fourier integrals*, **2** (Clarendon Press Oxford, 1948).
19. Diaz, R. E. & Alexopoulos, N. G. An analytic continuation method for the analysis and design of dispersive materials. *IEEE Trans. Antennas Propag.* **45**, 1602–1610 (1997).

**20.** https://www.nanolive.ch/.

**21.** https://www.tomocube.com/.

**22.** Tian, L. & Waller, L. Quantitative differential phase contrast imaging in an led array microscope. *Opt. Express* **23**, 11394–11403 (2015).

**23.** Zheng, G., Horstmeyer, R. & Yang, C. Wide-field, high-resolution Fourier Ptychographic microscopy. *Nat. Photonics* **7**, 739–745 (2013).

**24.** Sun, J. *et al.* High-speed fourier ptychographic microscopy based on programmable annular illuminations. *Sci. Rep.* **8**, 1–12 (2018).

**25.** Ling, R. *et al.* High-throughput intensity diffraction tomography with a computational microscope. *Biomed. Opt. Express* **9**, 2130 (2018).

**26.** Li, J. *et al.* High-speed in vitro intensity diffraction tomography. *Adv. Photonics* **1**, 066004 (2019).

**27.** Horstmeyer, R. *et al.* Diffraction tomography with fourier ptychography. *Optica* **3**, 827–835 (2016).

**28.** Kim, T. *et al.* White-light diffraction tomography of unlabelled live cells. *Nat. Photonics* **8**, 256–263 (2014).

**29.** Eckert, R., Phillips, Z. F. & Waller, L. Efficient illumination angle self-calibration in Fourier ptychography. *Appl. Opt.* **57**, 5434 (2018).

**30.** Hosseini, P. *et al.* Pushing phase and amplitude sensitivity limits in interferometric microscopy. *Opt. Lett.* **41**, 1656–1659 (2016).

**31.** Juffmann, T., de los Ríos Sommer, A. & Gigan, S. Local optimization of wave-fronts for optimal sensitivity phase imaging (lowphi). *Opt. Commun.* **454**, 124484 (2020).

**32.** Chandramohanadas, R. *et al.* Biophysics of malarial parasite exit from infected erythrocytes. *PloS One* **6**, e20869 (2011).

**33.** Baek, Y., Lee, K., Shin, S. & Park, Y. Kramers–kronig holographic imaging for high-space-bandwidth product. *Optica* **6**, 45–51 (2019).

**34.** Li, J. *et al.* Efficient quantitative phase microscopy using programmable annular led illumination. *Biomed. Opt. Express* **8**, 4687–4705 (2017).

**35.** Zheng, G., Horstmeyer, R. & Yang, C. Wide-field, high-resolution fourier ptychographic microscopy. *Nat. photonics* **7**, 739–745 (2013).

**36.** Chowdhury, S. *et al.* High-resolution 3d refractive index microscopy of multiple-scattering samples from intensity images. *Optica* **6**, 1211–1219 (2019).

**37.** Zhou, S. *et al.* Accelerated fourier ptychographic diffraction tomography with sparse annular led illuminations. *J. Biophotonics* e202100272 (2021).

**Table S1.** Comparison of different QPI and ODT techniques.

| Terms / Techniques | Data formulation | Raw data type | Imaging dimension | Source coherence and optical configuration | Scattering approximation | Key issues |
|---|---|---|---|---|---|---|
| DHM | Interferometric | Hologram (1 frame for off-axis) | 2D Phase | High (laser); Interferometric system (off-axis or on-axis) | | Speckle noise; Need phase unwrapping; K-K phase demodulation[33] |
| ODT | Interferometric | Hologram (∼ 200 frames) | 3D RI | High (laser); Interferometric system (off-axis or on-axis) | $1^{st}$ order Born or Rytov approximation | Mechanical angle scanning[6] |
| TIE | Non-interferometric | Through-focus intensity stack (3 frames) | 2D Phase | Low (LED); Can be partially coherent; Bright-field imaging system | | Carefully tuning of defocus distance and design of illumination pattern for robust phase TFs[10,34] |
| DPC | Non-interferometric | Diverse illumination intensity stack (4 frames) | 2D Phase | Low (LED); Can be partially coherent; Bright-field imaging system | | Need matched illumination condition and design of illumination pattern for robust phase TFs[22] |
| K-K QPI | Non-interferometric | Diverse illumination intensity stack (∼ 6 frames) | 2D Phase | Low (LED); Bright-field imaging system | | Need matched illumination condition; 2D K-K phase demodulation[17] |
| FPM | Non-interferometric | Diverse illumination intensity stack (∼ 200 frames) | 2D Phase | Low (LED); Bright-field imaging system | | Need matched illumination condition and iterations for phase reconstruction; Large amount of darkfield dataset[4,35] |
| IDT | Non-interferometric | Diverse illumination intensity stack (∼ 200 frames) | 3D RI | Low (LED); Bright-field imaging system | $1^{st}$ order Born approximation or multiple scattering | Need matched illumination condition[17]; Iterative algorithm for multiple scattered situation[36] |
| FPDT | Non-interferometric | Diverse illumination intensity stack (∼ 3,000 frames) | 3D RI | Low (LED); Bright-field imaging system | $1^{st}$ order Born or Rytov approximation | Need matched illumination condition and iterations for 3D reconstruction[37]; Large amount of darkfield dataset[3] |
| PRISM | Non-interferometric | Through-focus intensity stack (∼ 8 frames) | 3D Phase | Low (LED); Polychromatic coherent; Bright-field imaging system | $1^{st}$ order Born approximation | No matched illumination condition required; Non-incoherent diffraction limit[13] |
| K-K DT | Non-interferometric | Diverse illumination intensity stack (∼ 30 frames) | 3D RI | Low (LED); Bright-field imaging system | $1^{st}$ order Born or Rytov approximation | Need matched illumination condition; 2D K-K phase demodulation[17] |
| TIDT-NSA | Non-interferometric | Both diverse illumination and through-focus intensity stack (204 frames) | 3D RI | Low (LED); Bright-field imaging system | $1^{st}$ order Born or Rytov approximation | No matched illumination condition required; 3D K-K phase demodulation |

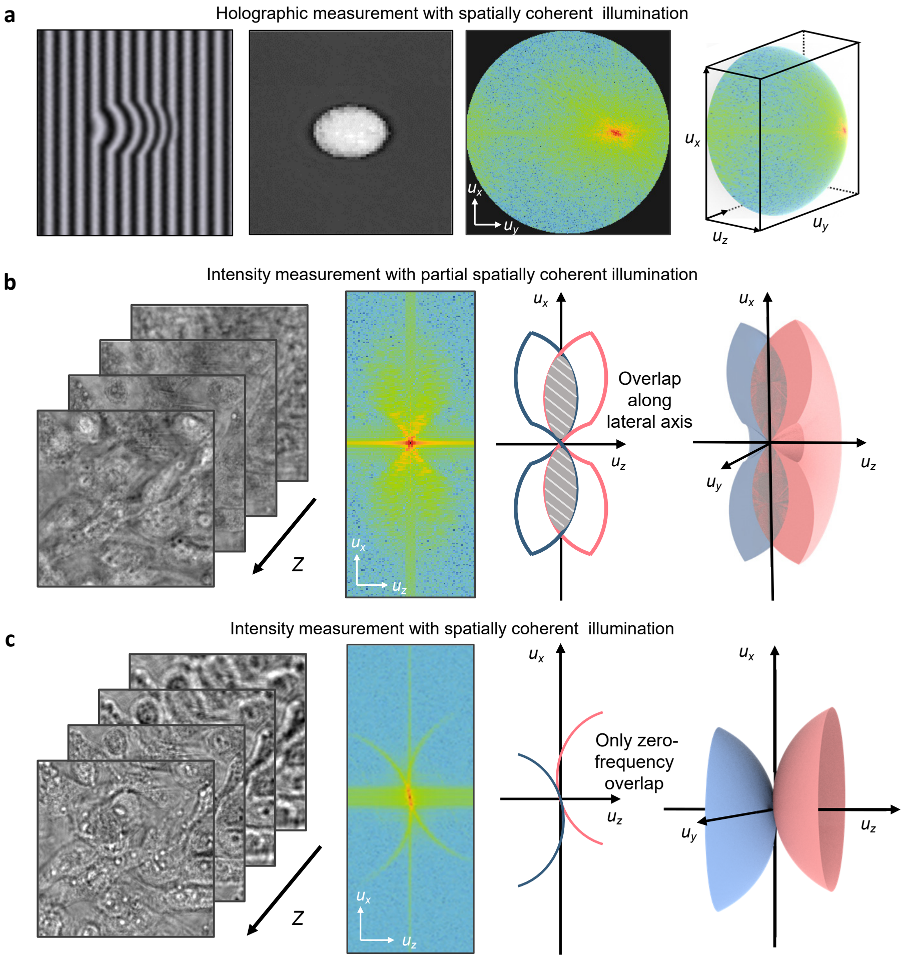


**Figure S1.** Illustration of basic principles of holographic diffraction tomography and noninterferometic transport of intensity diffraction tomography. a. Holographic measurement with coherent illumination. b,c. 3D intensity measurement with partially coherent illumination and coherent illumination.

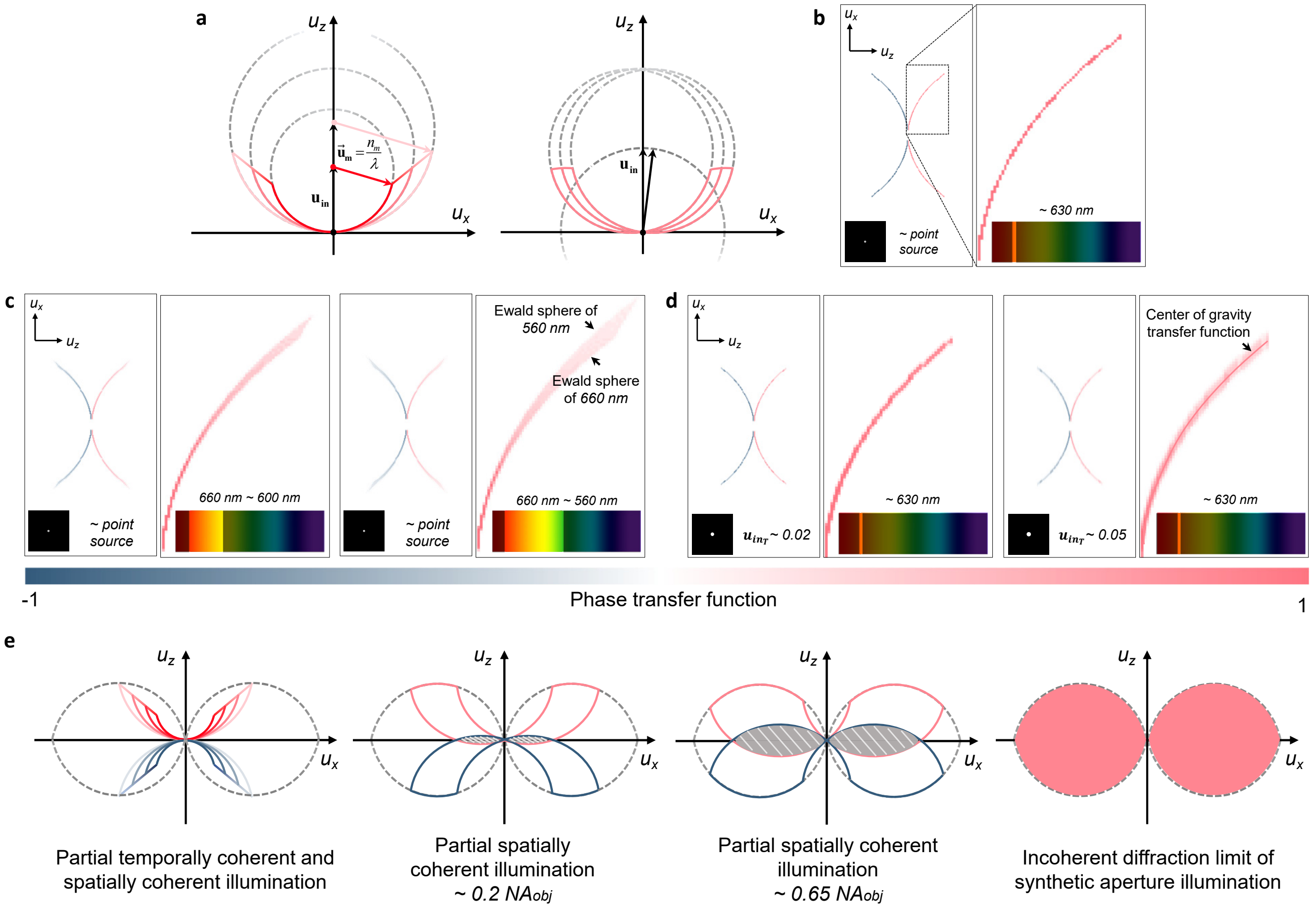


**Figure S2.** Effect of source temporal and spatial coherence on phase transfer function. a. Transfer function synthesis of temporal coherence (polychromatic source) and spatial coherence (extended source). b. Phase transfer function corresponding to a quasi-monochromatic ($\lambda$ = 630 nm), spatially coherent source. c. Phase transfer function distributions corresponding to spatially coherent polychromatic illuminations of different spectrum width. d. Phase transfer function distributions corresponding to quasi-monochromatic extended sources of different sizes. e. Comparison of phase transfer functions of different types of illuminations with different spatiotemporal coherence.

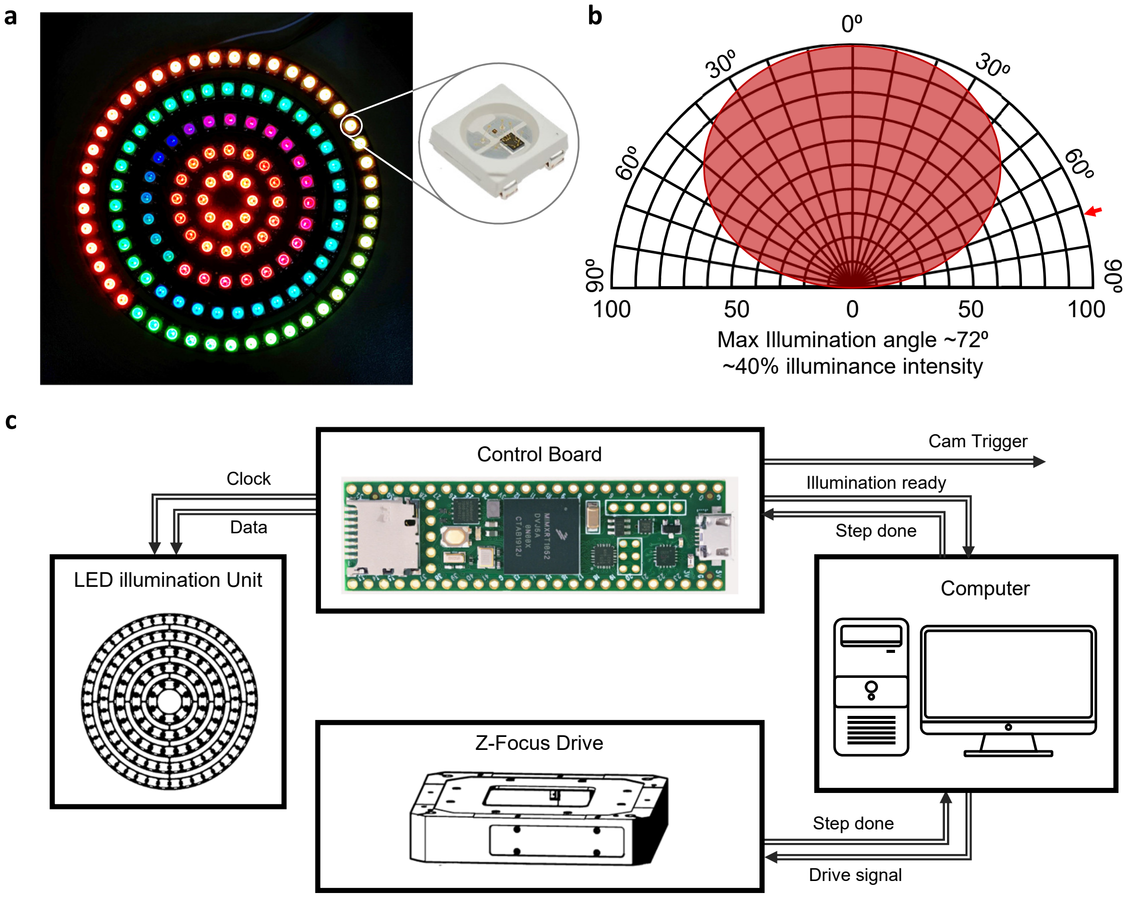


**Figure S3.** Hardware platform for TIDT-NSA and the diagram of electromechanical system synchronization. a. Photos of multi-annular programmable LED source and single LED element. b. Viewing angle and relative luminosity of LED illumination. LED can provide $\sim$ 40% illuminance intensity at the illumination NA 0.95 ($\sim$ 72 $^{\circ}$). c. Diagram of electromechanical system synchronization and instruction flow.

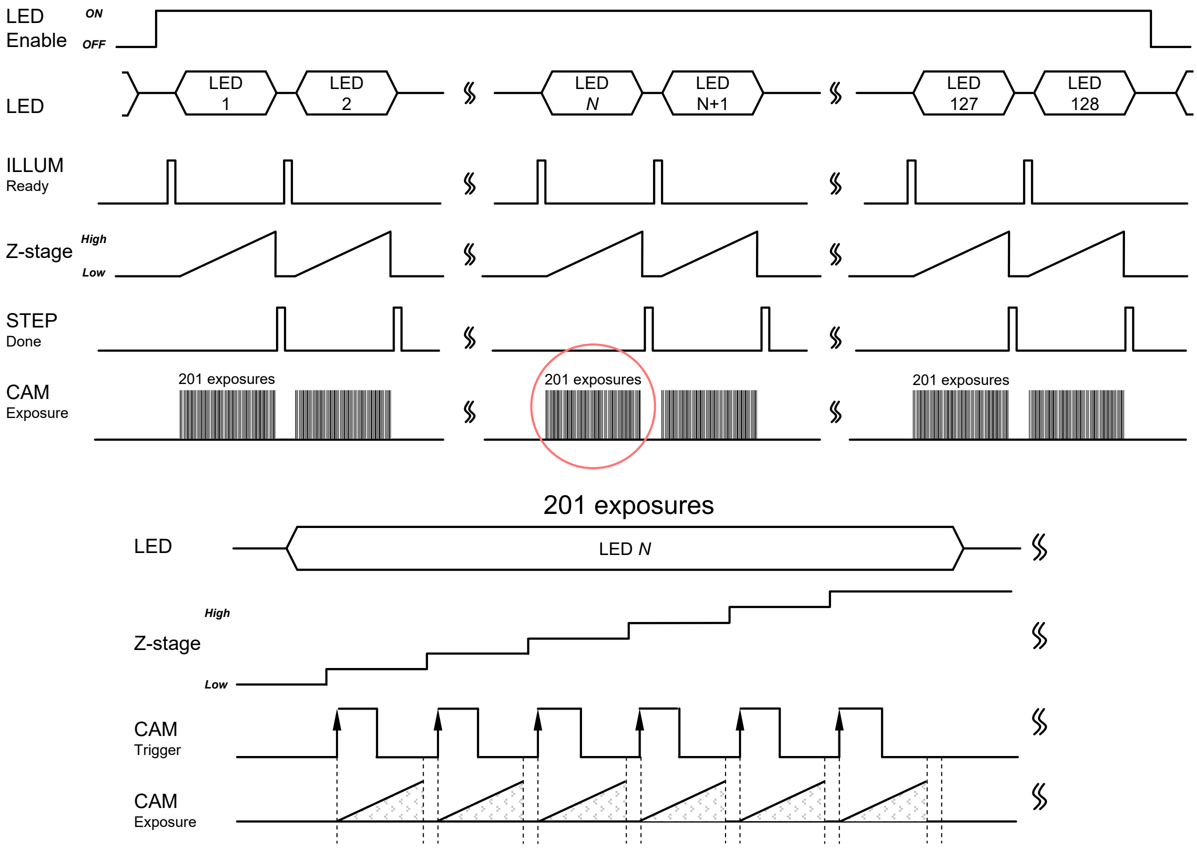


**Figure S4.** Time sequence of synchronization for one cycle of TIDT-NSA data acquisition. In the acquisition cycle, 128 intensity stacks are recorded during the on state of LED enable signal, and the acquisition of each intensity stack followed by the flag trigger of illumination ready. Top inset: Z-stage driving signals to scan the object along *z* direction from low position to high position during the acquisition of intensity stack at 201 different axial planes, and the step done signal indicate the acquisition complete of each stack. Bottom inset: Camera exposure sequence in each stack synchronized with stage movement containing 201 camera rising edge trigger, as illustrated in the red circle.

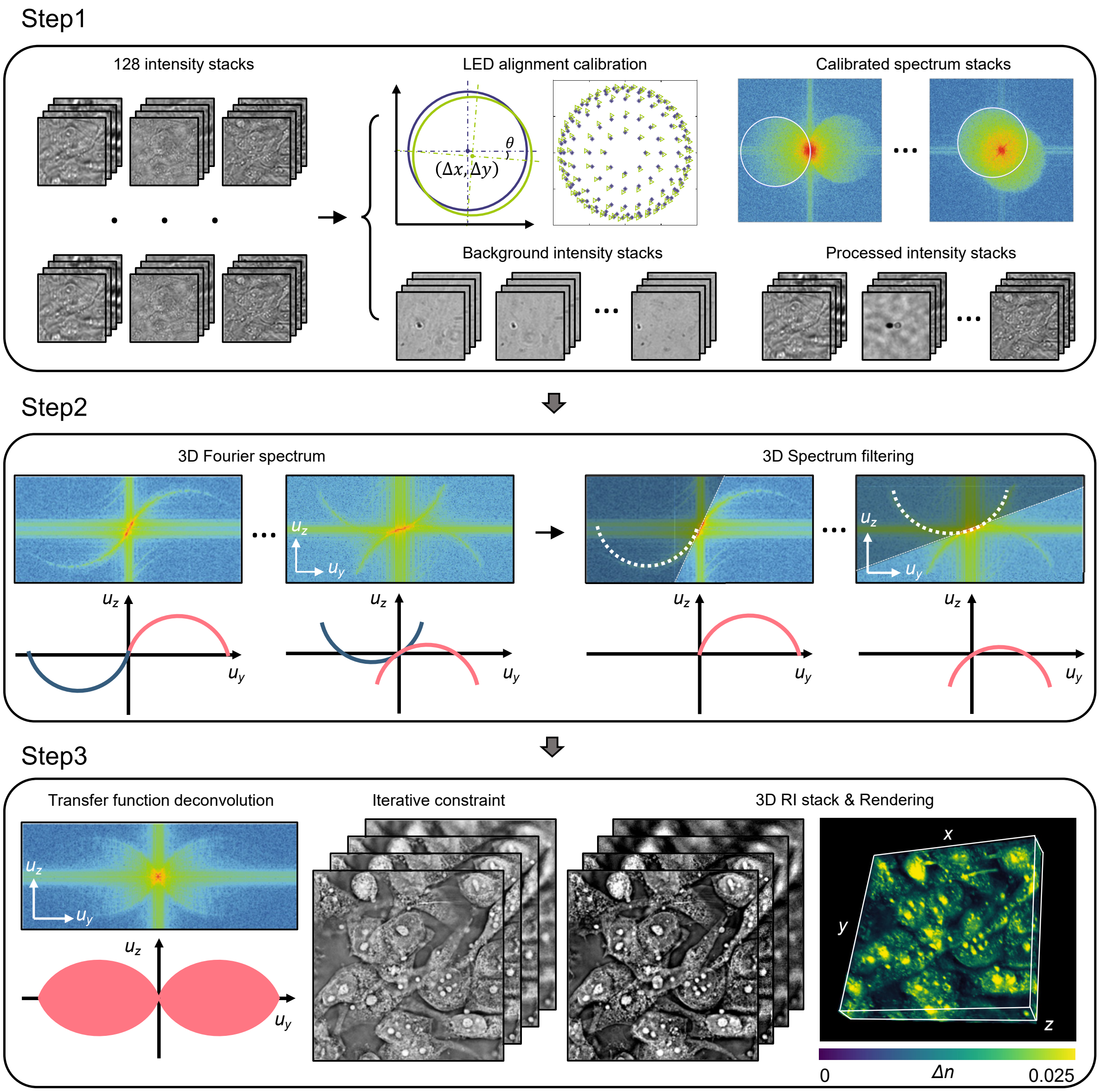


**Figure S5.** Pipeline of TIDT-NSA reconstruction algorithm. We calibrated the illumination angles with algorithmic self-calibration method for finely tuning the LED positions and recorded one set of background stacks in the absence of sample. After the intensity stack background removing, we perform the 3D half-space filtering on the captured Fourier spectrum and the single side-band spectrum, both containing real and imaginary parts of complex function. Implementing synthesized 3D transfer function deconvolution and iterative constraint algorithm to the 3D synthetic spectrum for final 3D RI distribution and rendering.

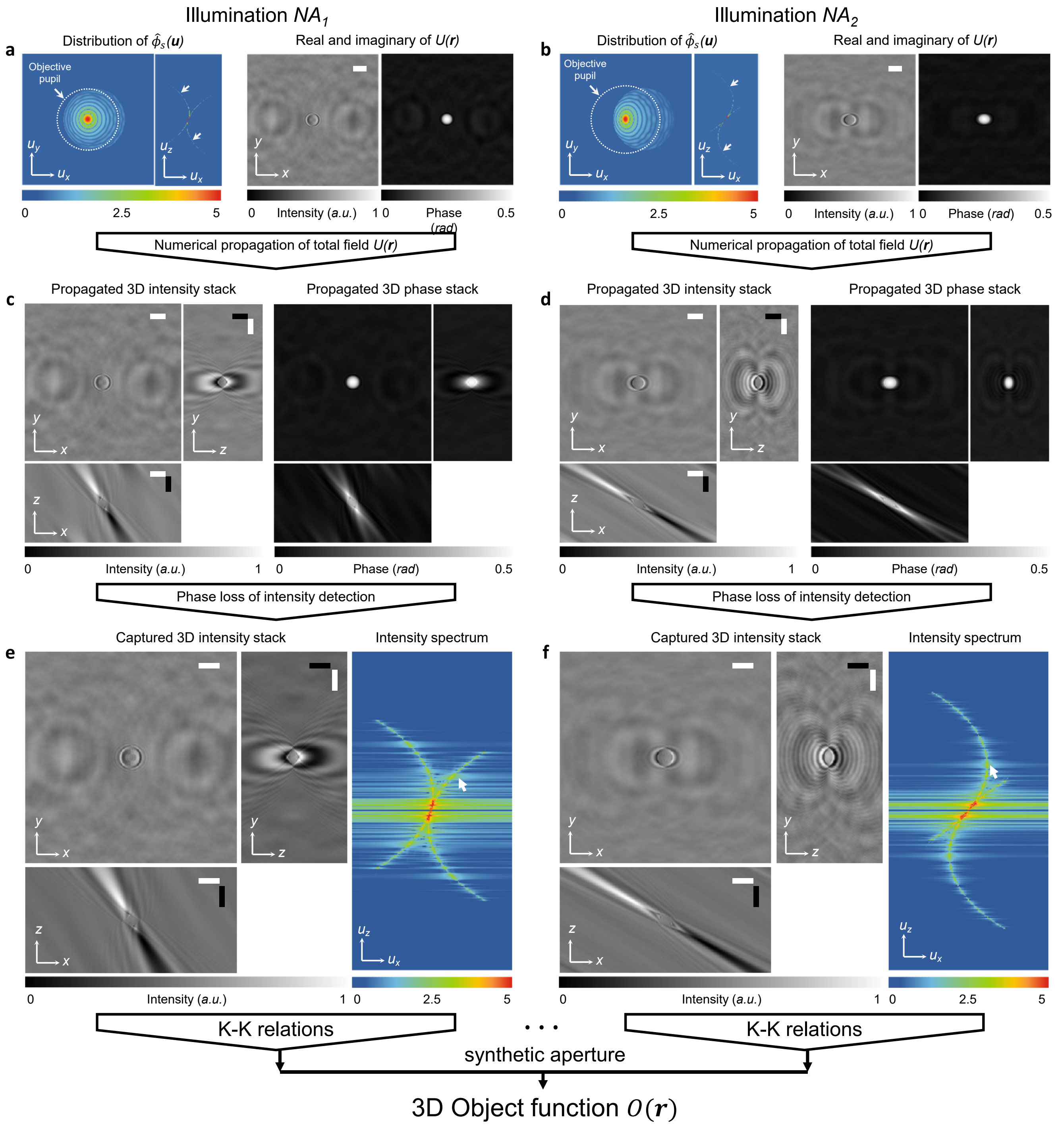


**Figure S6.** Simulations of a pure phase bead on 3D scattering and 2D numerical propagation under different illumination conditions. a,b. Distribution of 2D complex phase function spectrum $\hat{\phi}_s(\mathbf{u})$ from 3D Ewald sphere and real and imaginary parts of complex amplitude function $U(\mathbf{r})$. c,d. Propagated 3D intensity and phase stacks of complex amplitude through numerical propagation. e,f. Simulated captured 3D intensity stack due to the phase loss of intensity detection. Scale bars: 5 $\mu$m.

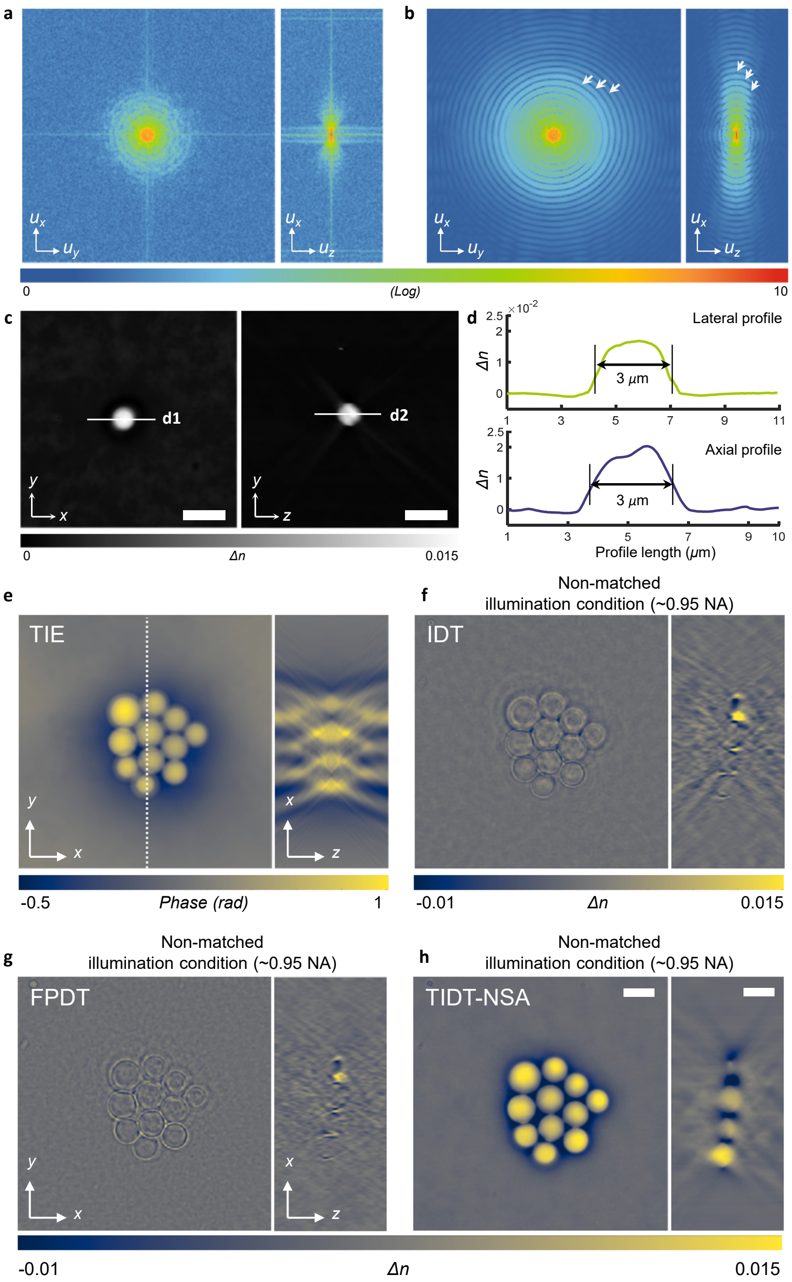


**Figure S7.** Experimental results of 3D tomographic RI of single polystyrene bead. a,b. Raw synthetic lateral and axial sections of spectrum and the constrained spectrum after the algorithms with non-negative and total variation regularization. c. RI tomograms of a single polystyrene bead with iterative constraint in the $x-y$ and $y-z$ planes. d. Two line profiles across the bead are indicated the lateral and axial reconstruction. e. Quantitative phase distributions of bead cluster along lateral and axial directions retrieved by TIE. f-h. Tomographic results of polystyrene beads utilizing IDT, FPDT, and TIDT-NSA under non-matched illumination condition (0.95 $NA_{ill}$ to 1.4 $NA_{obj}$). Scale bars: 5 $\mu$m.

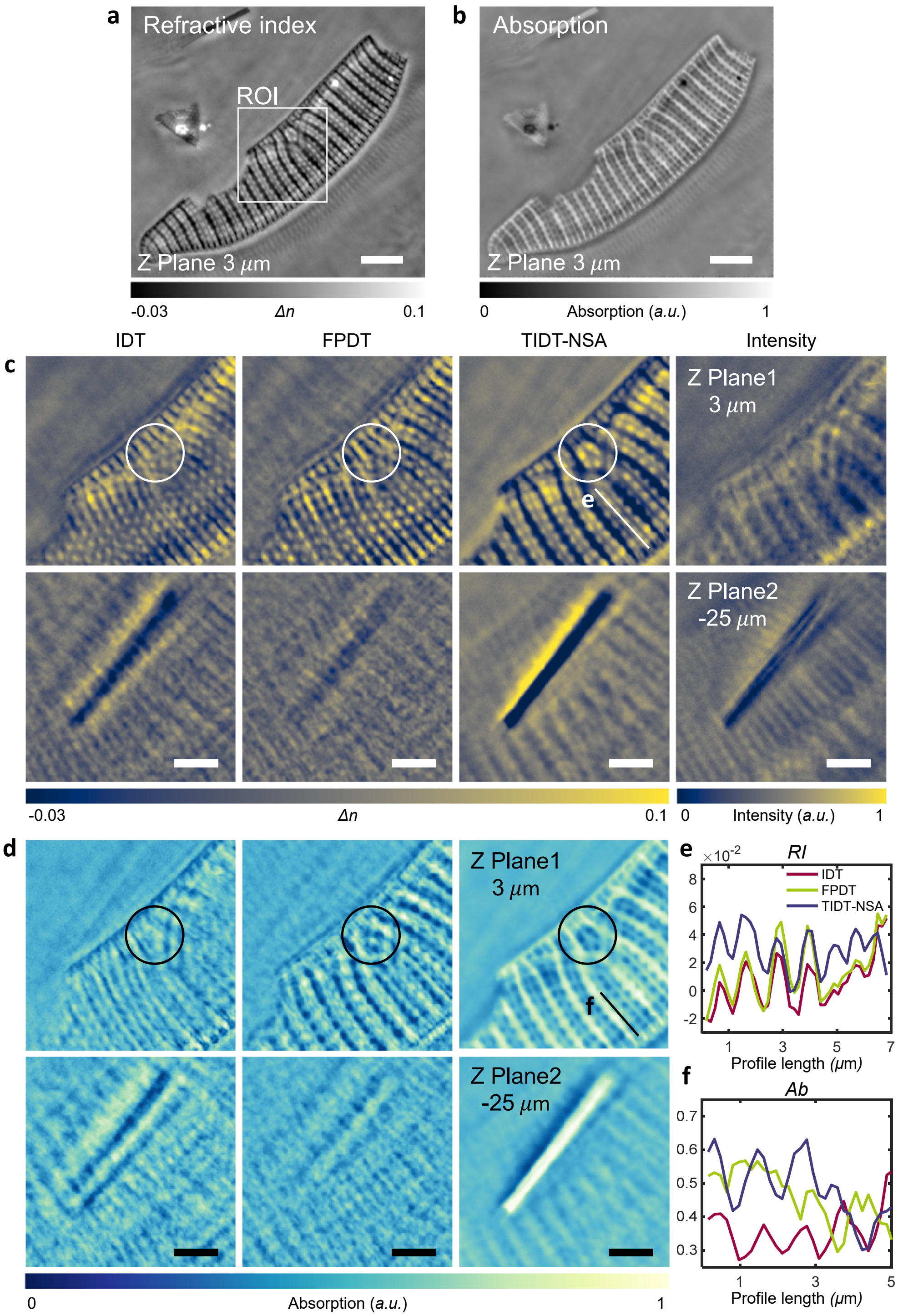


**Figure S8.** Benchmarking results of TIDT-NSA against other 3D phase imaging methods (IDT and FPDT) based on unicellular diatom microalgae under matched illumination condition (both 0.95 $NA_{ill}$ and $NA_{obj}$). a,b. Whole view of both 3D RI and absorption recovered by TIDT-NSA. c,d. Raw intensity, absorption, and RI distributions of an example region of interest image at two different planes. e,f. Two comparison line profiles across the diatom are indicated the lateral complex RI reconstruction by different tomographic methods. Scale bars: (a, b) 50 $\mu$m and (c, d) 10 $\mu$m.

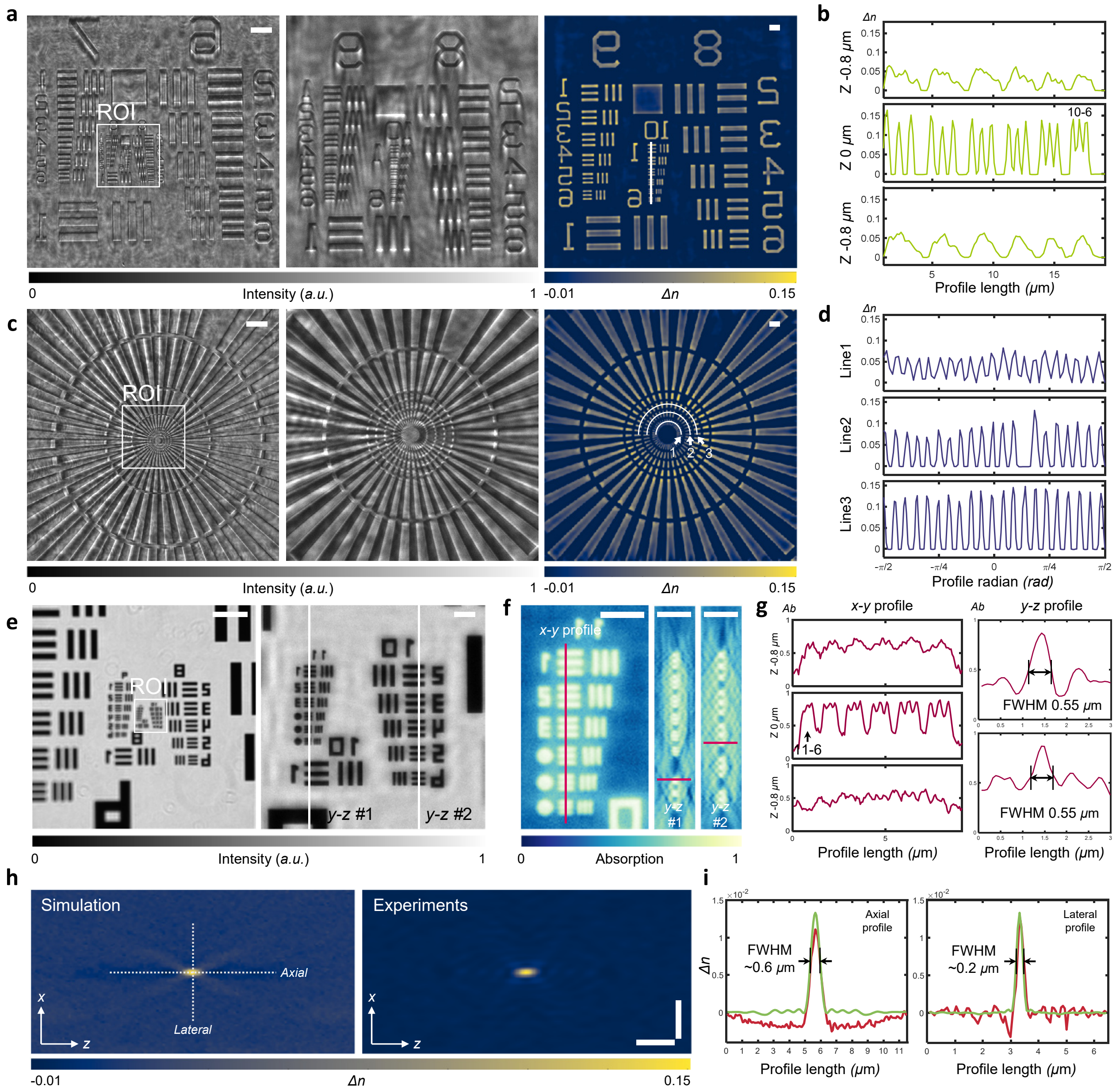


**Figure S9.** Resolution benchmarking of TIDT-NSA with both phase and absorption resolution target and nano-sphere. a-d. Experimental results of phase resolution target both on USAF pattern (minimal bar 10-6) and Siemens star pattern utilizing NA 0.95 objective and red light (wavelength $\lambda$ = 629 nm). e-g. Tomographic RI reconstruction of absorption positive USAF target which provides the maximum theoretical lateral resolution 274 nm (minimal bar 11-6 in USAF target) and axial resolution 0.55 $\mu$m in accordance with the theoretical resolution prediction. h,i. Experimental and simulated PSF of 100 nm diameter nano-sphere satisfying sub-diffraction sized object for characterization of 3D RI tomographic ability and imaging resolution. Scale bars: Full FOV of (a, c, e) 20 $\mu$m and ROI of (a, c, e), f, and h 3 $\mu$m.

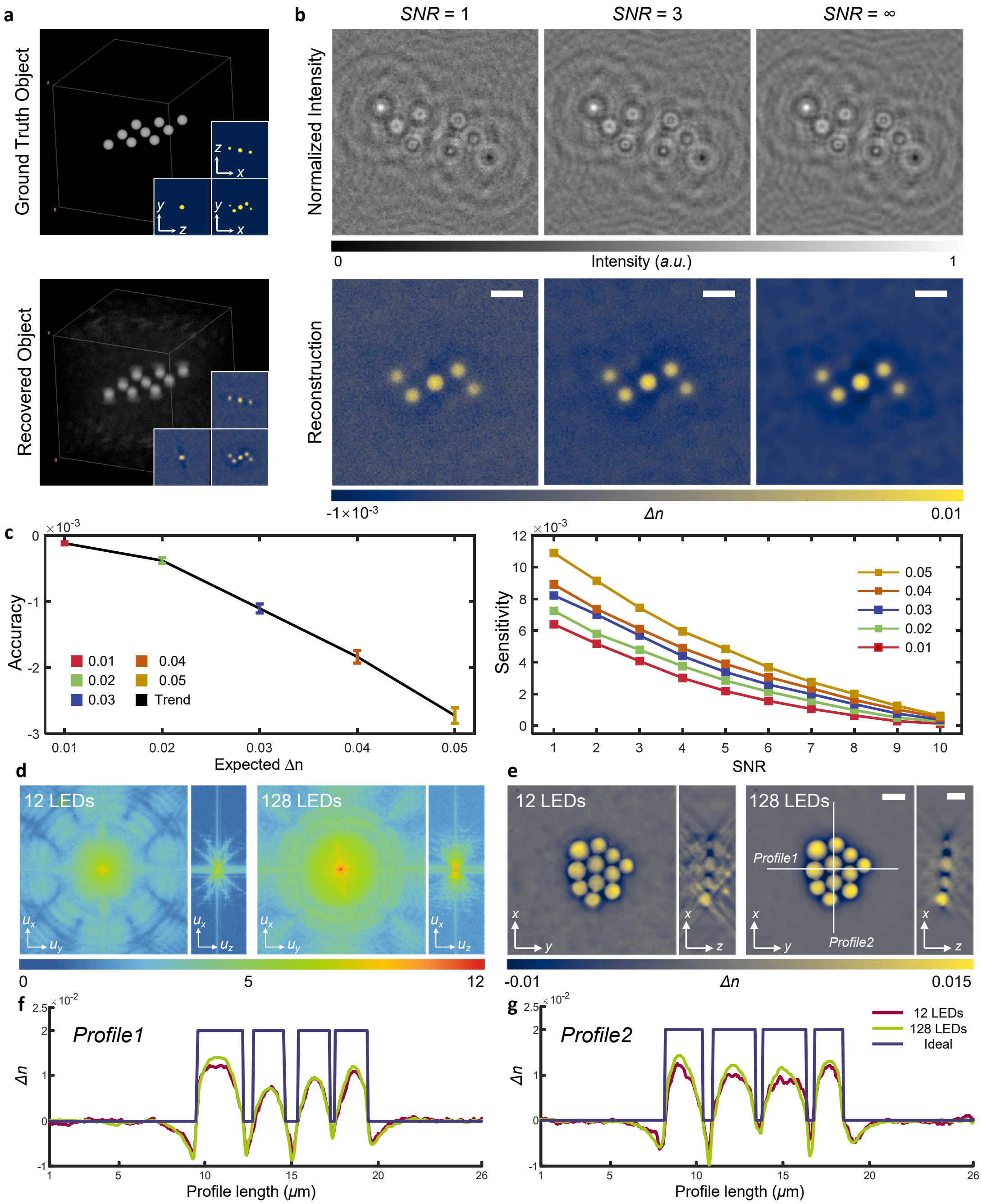


**Figure S10.** Simulation for quantifying TIDT-NSA accuracy and sensitivity, and quantitative comparison about the reconstructed RI results between the cases of full sampled and down-sampled LED illumination. a. Top: The object consists of a sphere array occupying a $33 \times 33 \times 33\ \mu m^3$ volume. Bottom: An example TIDT-NSA reconstruction. b. Simulated intensity images with different SNR at the in-fucus plane and in-focus RI slice reconstructions of TIDT-NSA. The median recovered RI over the sphere area compared with the ground truth object to determine the reconstruction accuracy and sensitivity. c. TIDT-NSA accuracy evaluations across the object RI and sensitivity analysis as a function of SNR. d-g. Quantitative comparison about the recovered Fourier spectrums and 3D RI slices of a cluster of polystyrene beads between the cases of 128 LED elements and 12 LED elements (10-fold less illumination number). Scale bars: 5 $\mu$m.

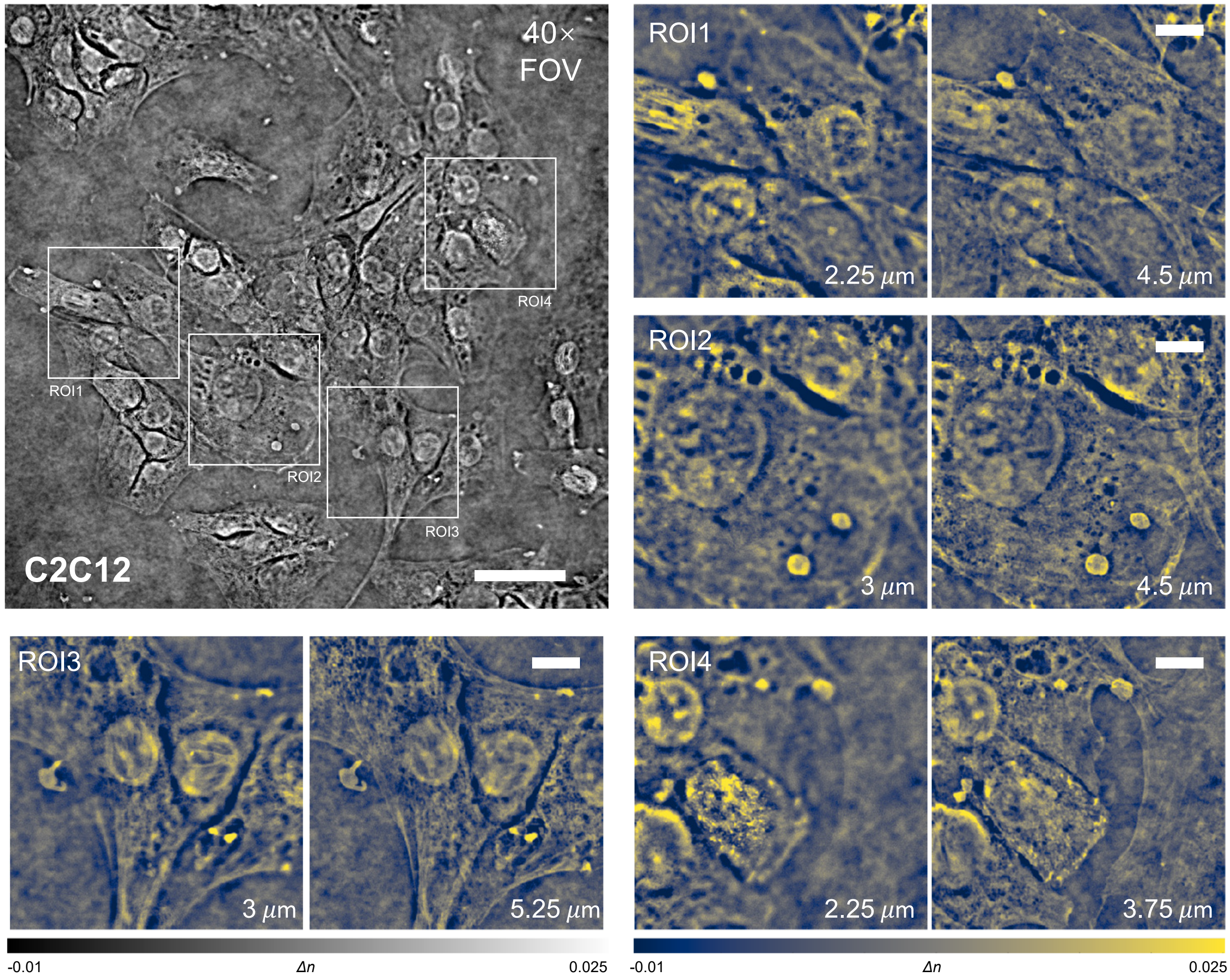


**Figure S11.** RI tomography of fixed C2C12 cell clusters imaged with 40× NA 0.95 objective, and $x-y$ cross-sectional views of four different tomogram ROIs at different axial planes. Scale bars: 15 $\mu$m.

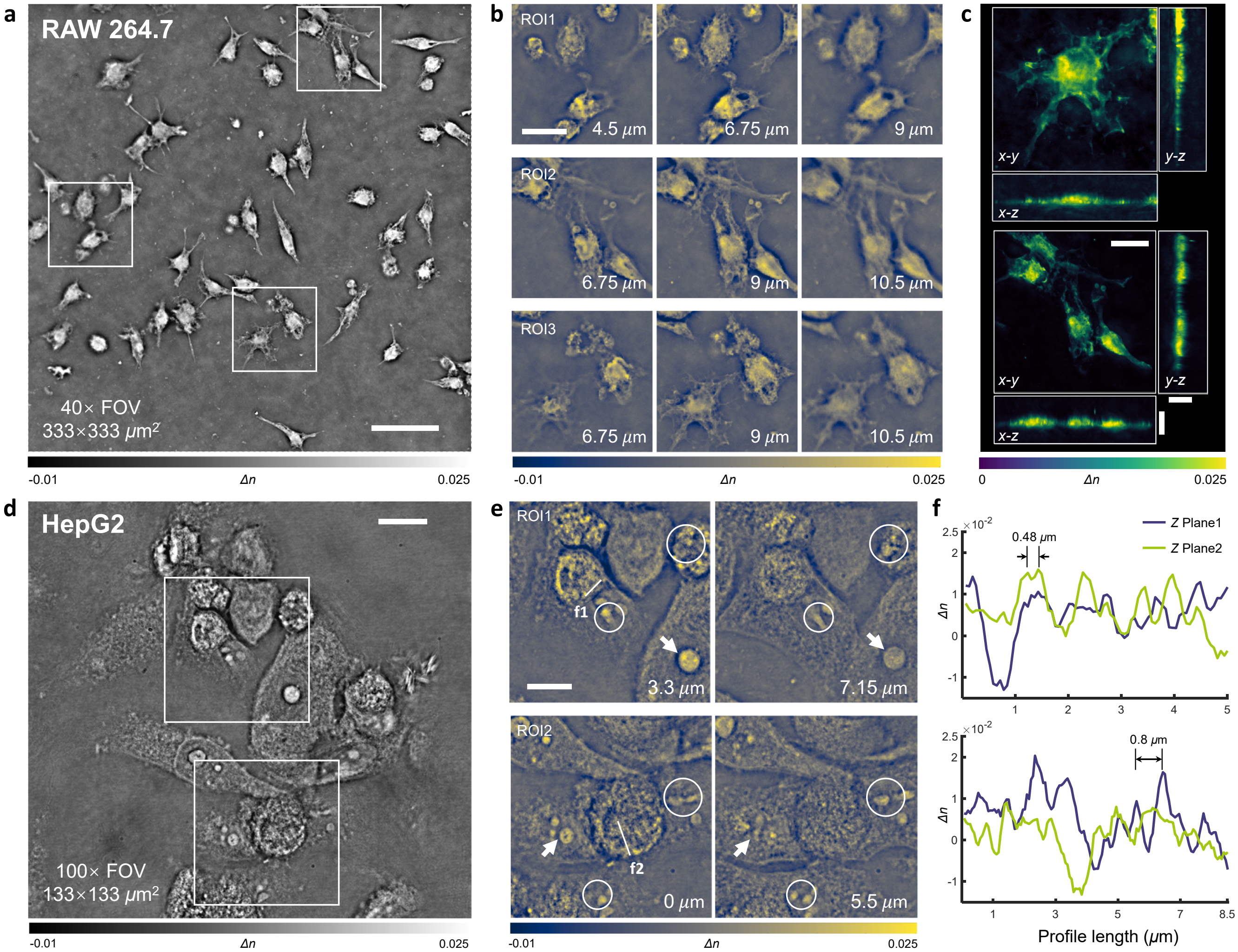


**Figure S12.** RI tomography of fixed RAW 264.7 and HepG2 cell clusters with different objective lenses. a-b. Fixed RAW 264.7 cells imaged with 40× objective over full FOV of 333 × 333 $\mu$m$^2$ and three enlarged $x-y$ ROIs of RI images at different axial planes. c. Maximum intensity projections volume-rendered 3D RI distributions of one single cell from ROI3 and the small cell cluster from ROI2 of RAW 264.7. d-e. Full FOV of HepG2 cells imaged with 100× NA 1.4 oil-immersion objective, and $x-y$ cross-sectional view of the tomogram ROIs at different axial planes. f. RI line profiles across two cells at two different axial planes in (e). Scale bars: (a) 50 $\mu$m and (b, c, d, e) 15 $\mu$m.

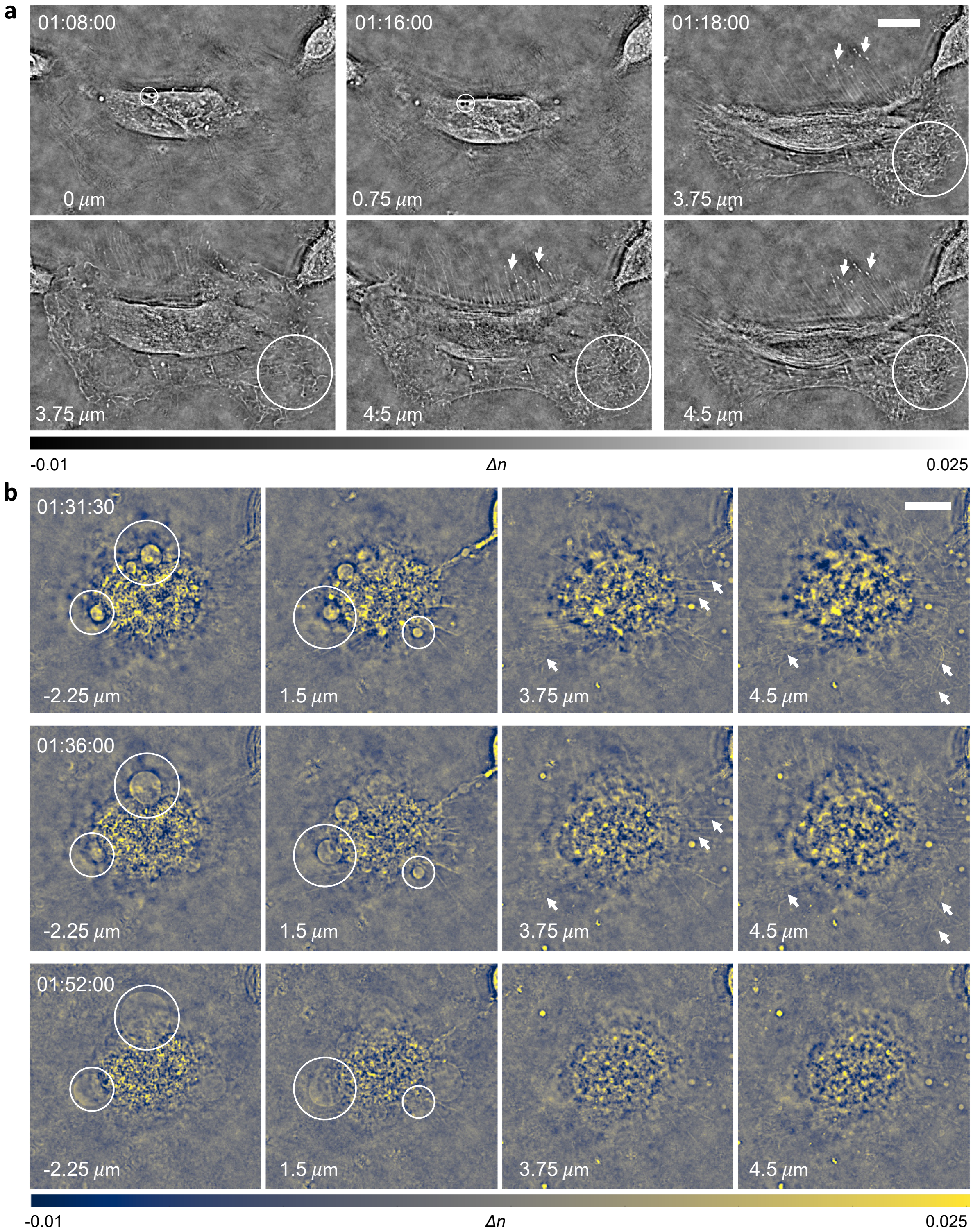


**Figure S13.** Cross-sectional view of the RI tomogram of HeLa cell apoptosis process. a. Recovered RI slices of HeLa cells at different time points and axial positions before cell volume shrinkage and structural collapse. b. RI distribution of HeLa cells at late stage of apoptosis. The volume of cell shrunk, fragments fall off from the surrounding structure, and the structure similar to bubbles and fine fibers indicated by arrows and circles. Scale bars: 20 $\mu$m.

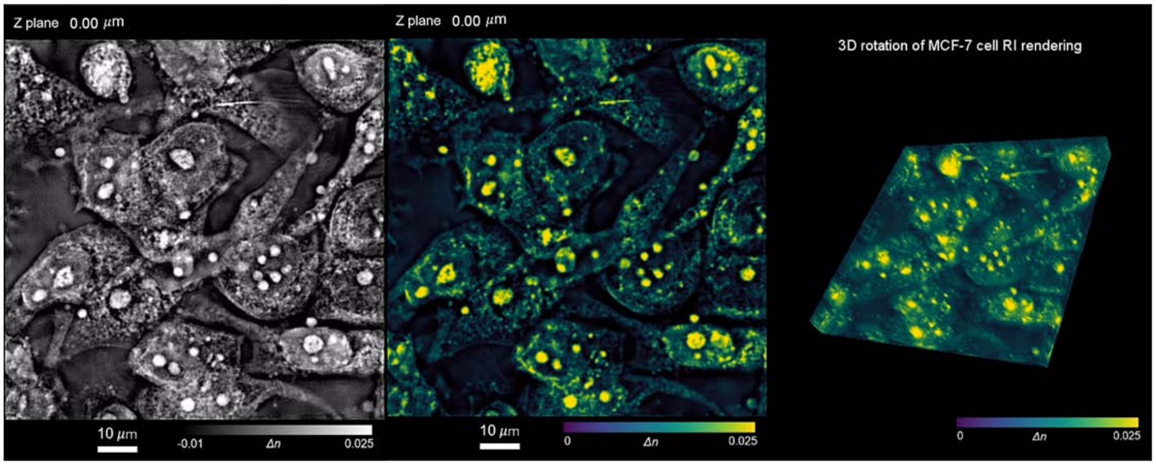


**Video S1.** RI change at different axial planes and 3D rotation of MCF-7 cell RI rendering.

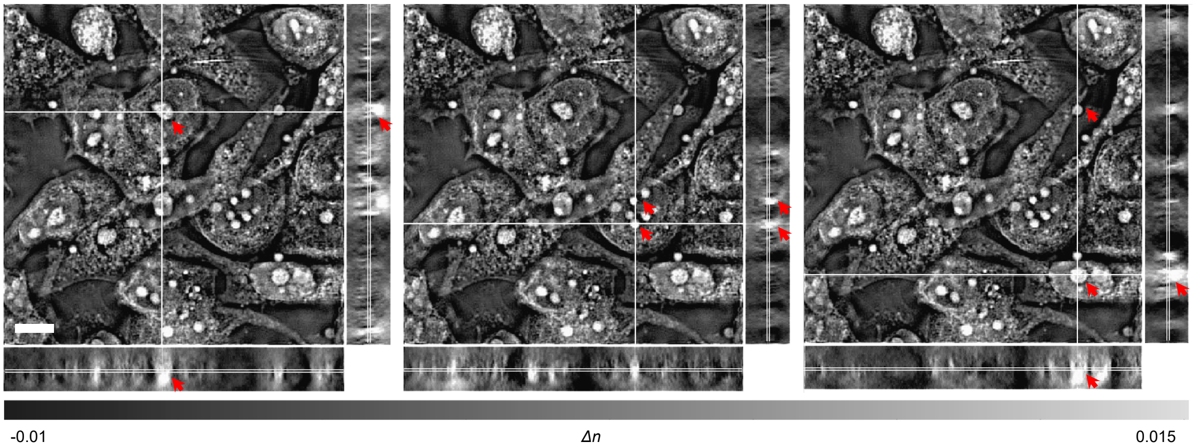


**Video S2.** RI views in three orientations simultaneously at different lateral positions of MCF-7 cells.

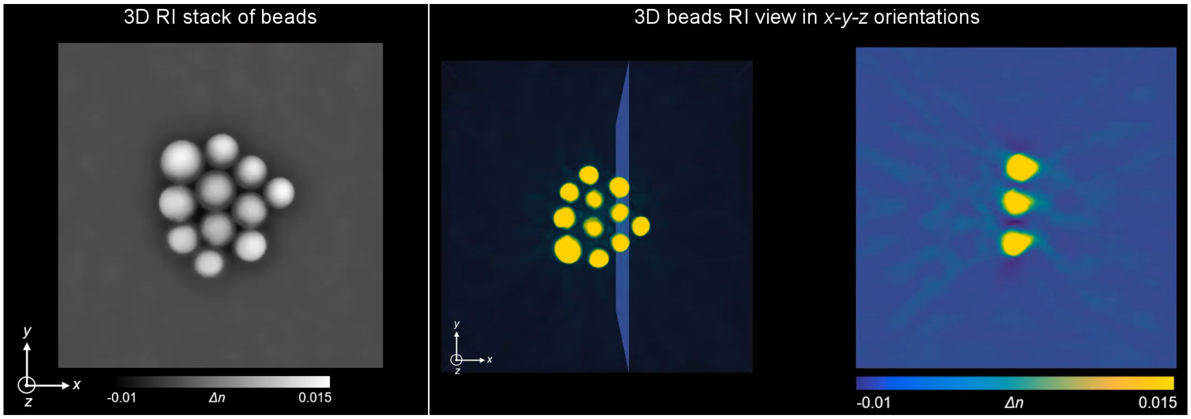


**Video S3.** 3D RI stack of beads and RI views in $x-y-z$ orientations.

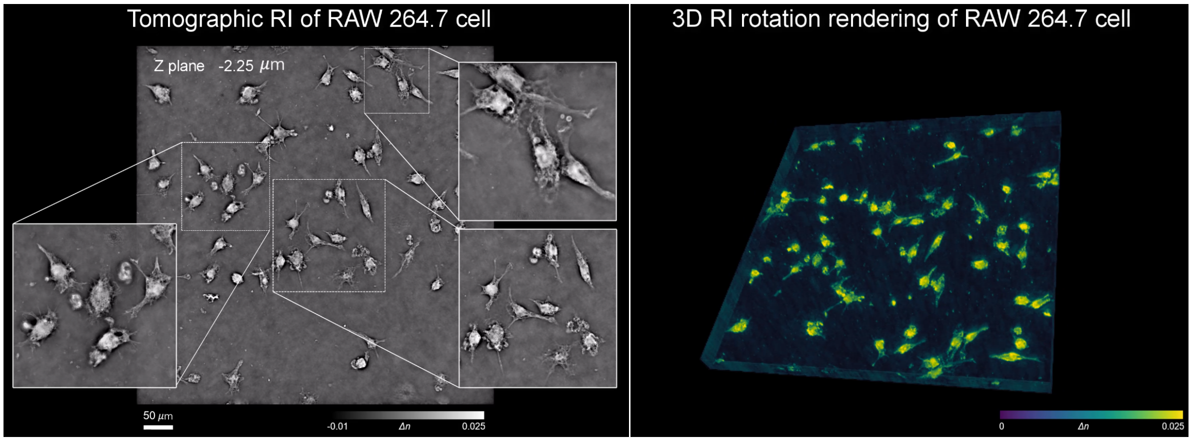


**Video S4.** Tomographic RI and 3D RI rotation rendering of RAW 264.7 cell.

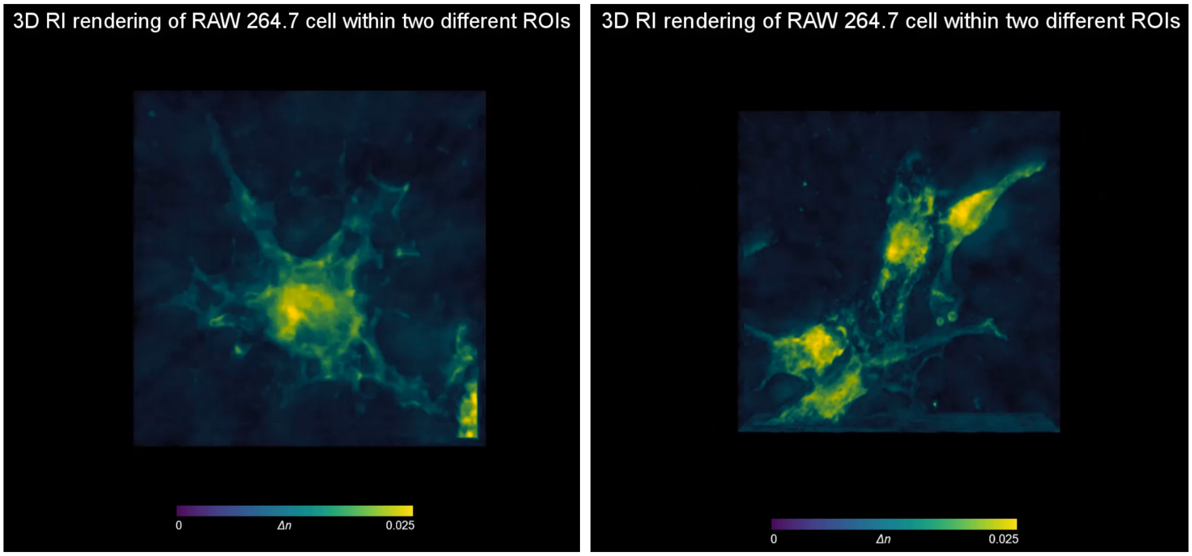


**Video S5.** 3D RI rendering of RAW 264.7 cell within two different ROIs.

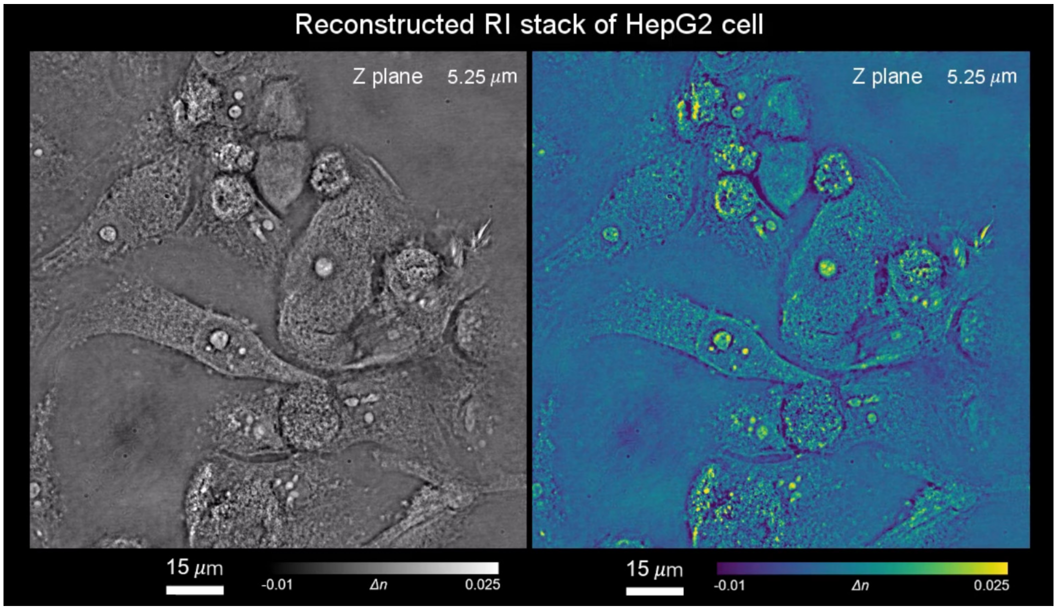


**Video S6.** Reconstructed RI stack of HepG2 cell.

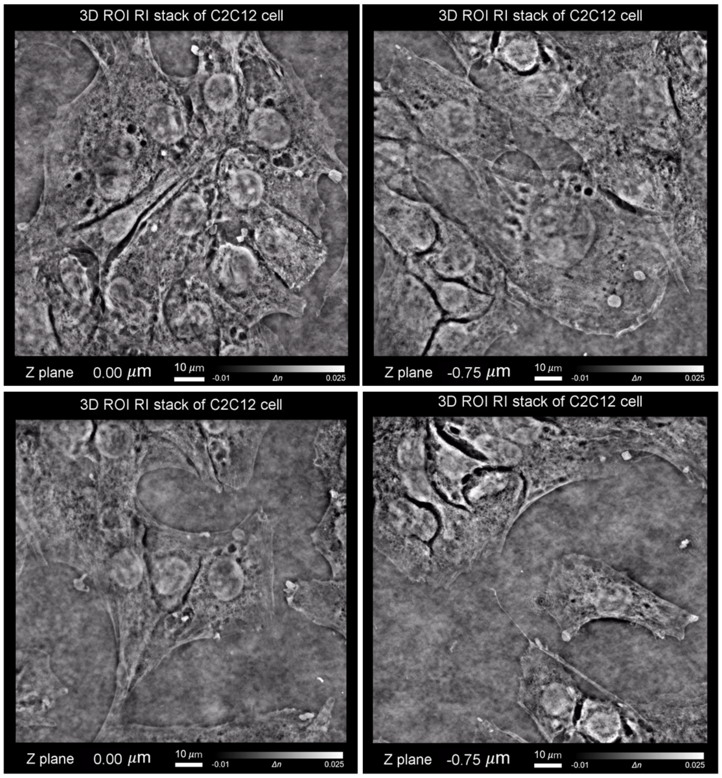


**Video S7.** 3D ROIs RI stack of C2C12 cell.

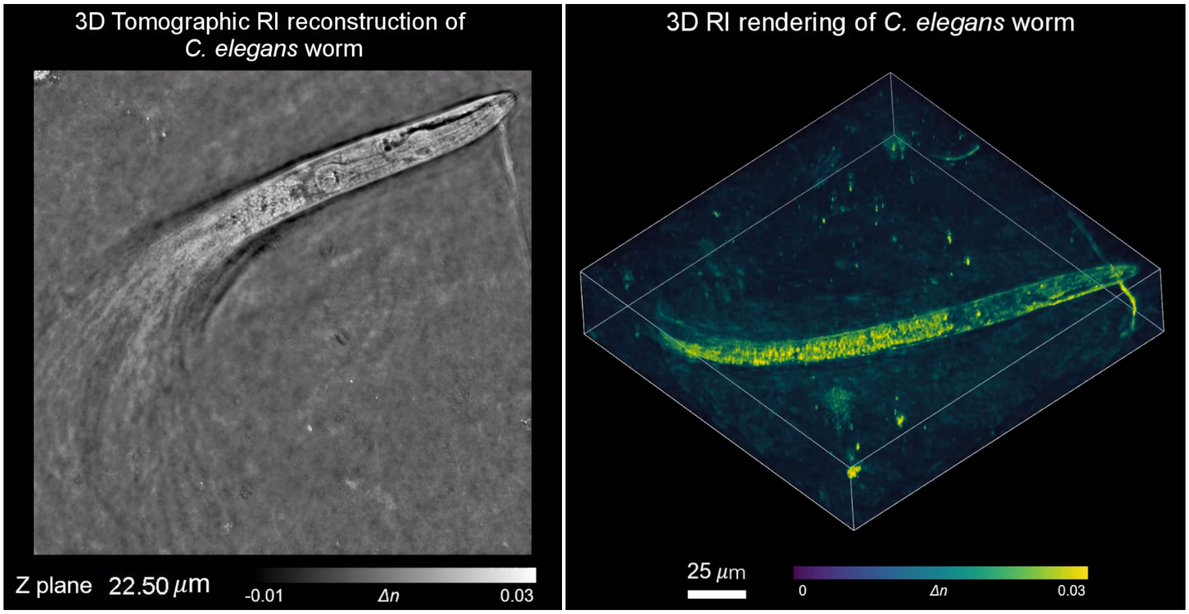


**Video S8.** 3D tomographic RI reconstruction and 3D RI rendering of *C. elegans* worm.

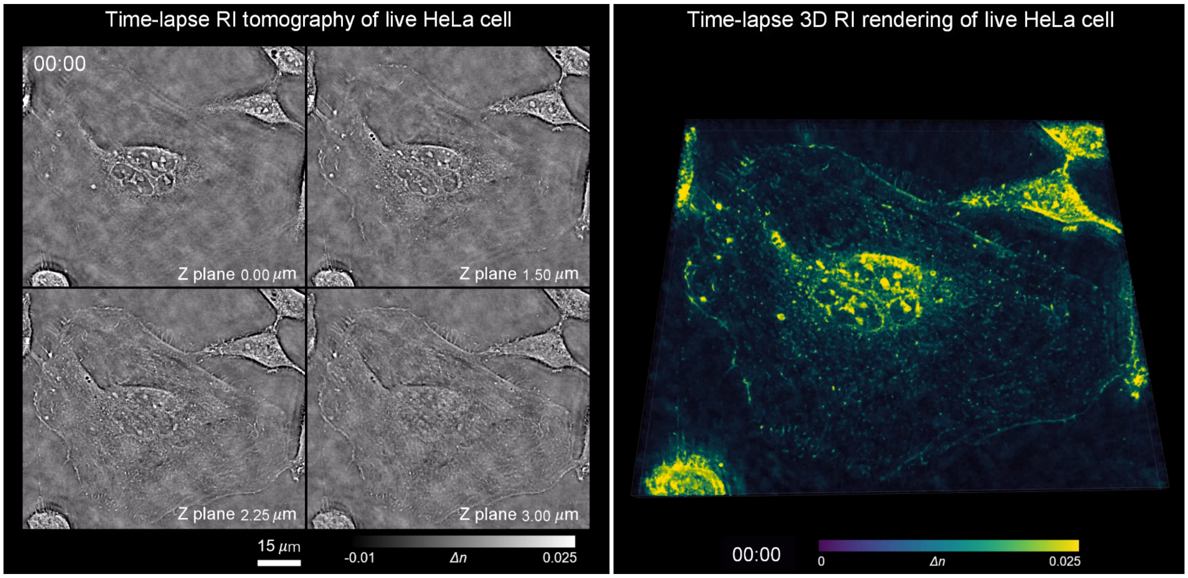


**Video S9.** Time-lapse tomographic RI and 3D time-lapse RI rendering of live HeLa cell.

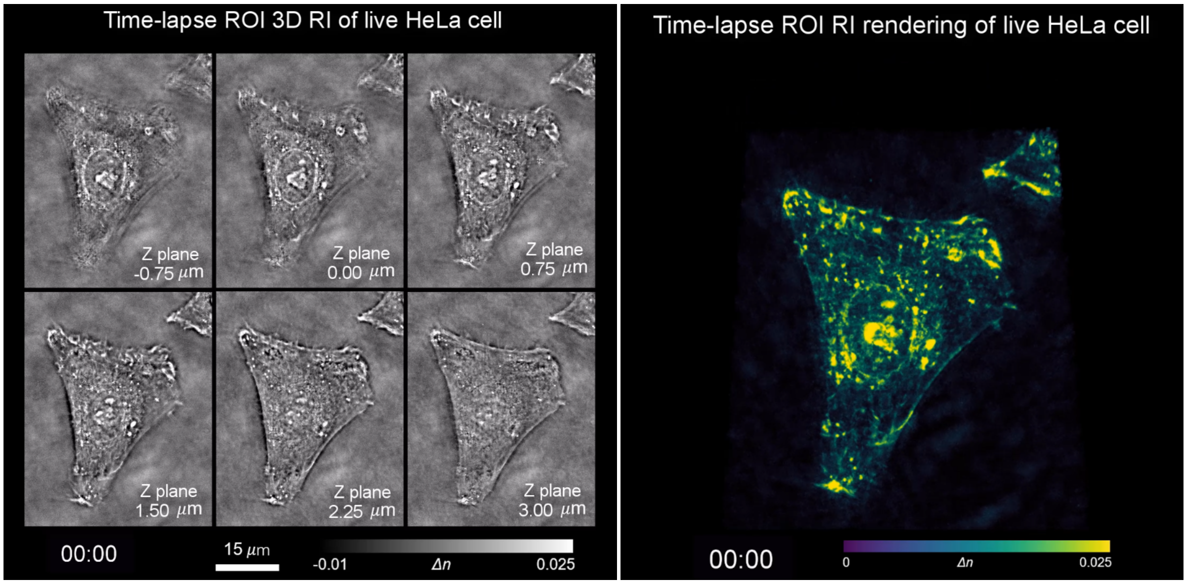


**Video S10.** Time-lapse 3D ROI RI stack and rendering of live HeLa cell.

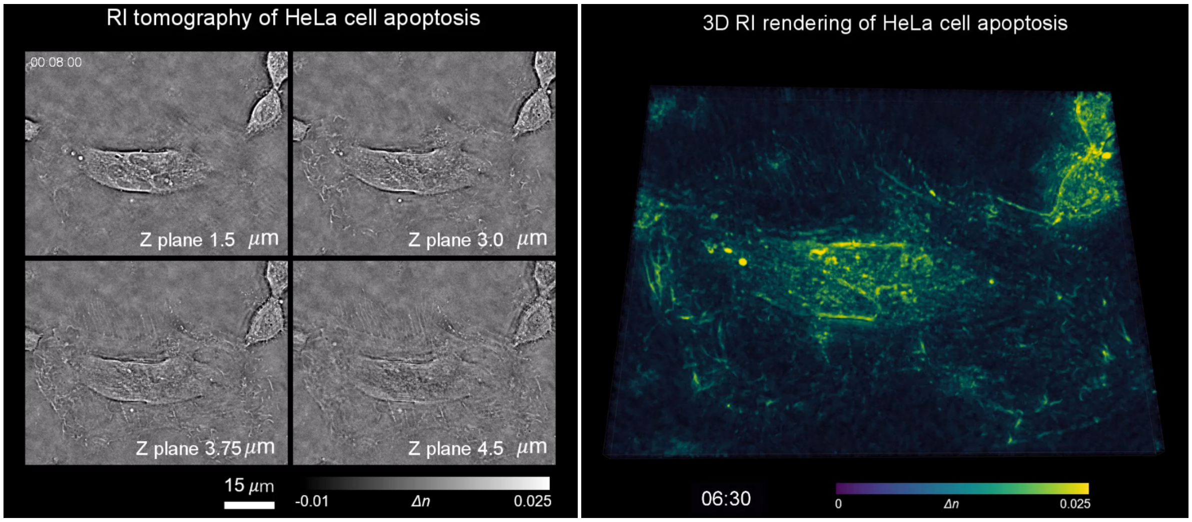


**Video S11.** RI tomography and 3D RI rendering of HeLa cell apoptosis.
